# Optimal Recall from Bounded Metaplastic Synapses: Predicting Functional Adaptations in Hippocampal Area CA3

**DOI:** 10.1371/journal.pcbi.1003489

**Published:** 2014-02-27

**Authors:** Cristina Savin, Peter Dayan, Máté Lengyel

**Affiliations:** 1 Computational & Biological Learning Lab, Department of Engineering, University of Cambridge, Cambridge, United Kingdom; 2 Gatsby Computational Neuroscience Unit, University College London, London, United Kingdom; Indiana University, United States of America

## Abstract

A venerable history of classical work on autoassociative memory has significantly shaped our understanding of several features of the hippocampus, and most prominently of its CA3 area, in relation to memory storage and retrieval. However, existing theories of hippocampal memory processing ignore a key biological constraint affecting memory storage in neural circuits: the bounded dynamical range of synapses. Recent treatments based on the notion of metaplasticity provide a powerful model for individual bounded synapses; however, their implications for the ability of the hippocampus to retrieve memories well and the dynamics of neurons associated with that retrieval are both unknown. Here, we develop a theoretical framework for memory storage and recall with bounded synapses. We formulate the recall of a previously stored pattern from a noisy recall cue and limited-capacity (and therefore lossy) synapses as a probabilistic inference problem, and derive neural dynamics that implement approximate inference algorithms to solve this problem efficiently. In particular, for binary synapses with metaplastic states, we demonstrate for the first time that memories can be efficiently read out with biologically plausible network dynamics that are completely constrained by the synaptic plasticity rule, and the statistics of the stored patterns and of the recall cue. Our theory organises into a coherent framework a wide range of existing data about the regulation of excitability, feedback inhibition, and network oscillations in area CA3, and makes novel and directly testable predictions that can guide future experiments.

## Author Summary

Memory is central to nervous system function and has been a particular focus for studies of the hippocampus. However, despite many clues, we understand little about how memory storage and retrieval is implemented in neural circuits. In particular, while many previous studies considered the amount of information that can be stored in synaptic connections under biological constraints on the dynamic range of synapses, how much of this information can be successfully recovered by neural dynamics during memory retrieval remains unclear. Here, we use a top-down approach to address this question: we assume memories are laid down in bounded synapses by biologically relevant plasticity rules and then derive from first principles how the neural circuit should behave during recall in order to retrieve these memories most efficiently. We show that the resulting recall dynamics are consistent with a wide variety of properties of hippocampal area CA3, across a range of biophysical levels – from synapses, through neurons, to circuits. Furthermore, our approach allows us to make novel and experimentally testable predictions about the link between the structure, dynamics, and function of CA3 circuitry.

## Introduction

The hippocampus, together with associated medial temporal-lobe structures, plays a critical role in memory storage and retrieval. A venerable line of classical theoretical work has shaped our understanding of how different hippocampal subfields subserve this function [1], [2]. At the core of this body of work is the notion that area CA3 of the hippocampus operates as an autoassociator, retrieving previously stored memory traces from noisy or partial cues by the process of pattern completion. Furthermore, the theoretical framework of autoassociative memory networks helped elucidate how recurrently-coupled neural circuits, such as CA3 [3], are capable of such pattern completion [4]–[10]. In this framework, synaptic plasticity stores memory traces in the efficacies (or weights) of the recurrent synapses of the neural circuit, and the recall of memories is achieved by the dynamical evolution of network activity through the synapses that were previously altered [11]. This framework has paved the way for a thorough analysis of the memory capacity of recurrent neural circuits [4], [8]–[10], and ensuing experimental results have confirmed many of its qualitative predictions [12], [13]. However, despite much progress, existing models of auto-associative memories make drastic simplifying assumptions, as we describe below, concerning both the synaptic plasticity rules storing information in the circuit and the dynamics of the network at recall.

First, at the level of memory storage, one powerful, yet biologically untenable, simplification made by most existing models [4], [8]–[10], [14] is the use of additive learning rules, whereby the cumulative effect of storing multiple memory traces is obtained as the linear sum of the contributions made by storing each individual trace. This simplification makes the analysis of the circuit tractable and suggests high memory capacity, but it also implies that synaptic weights can grow arbitrarily large or even switch sign, thereby violating Dale's principle. The shortcomings of assuming additive learning rules can be partially alleviated by introducing additional mechanisms such as synaptic scaling or metaplasticity, that ensure synapses are maintained in the relevant biological range [15]. Metaplasticity is loosely defined as any mechanism that manipulates or modulates synaptic plasticity; it comes in many forms, from the sliding threshold in BCM-like models [16] to sophisticated cascade models [17]. It is ubiquitous in the neocortex [18]–[20] and the hippocampus [21], and has long been implicated in endowing synapses with powerful computational properties [16]. Importantly for memory storage, it was shown that one particular form of metaplasticity, the cascade model [17], enables information to be stored in bounded synapses almost as efficiently as additive learning rules, whereas synapses with the same range of efficacies but without metaplasticity are hopelessly poor [17]. Unfortunately, despite their advantage at storing information, metaplastic synapses were found to perform equally poorly when the amount of recalled information was measured instead [22], indicating that much of the information laid down in the synapses remained inaccessible for the standard attractor dynamics used at retrieval. Thus, perhaps surprisingly, we still do not know how competent memory recall is possible from more realistic synapses that suffer from a bounded dynamical range.

Second, at the level of retrieval, there are also several aspects of hippocampal circuit dynamics of which we lack a theoretical account. For example, experimental work has long shown that synaptic plasticity is accompanied by changes in the excitability of CA3 neurons [23]–[25], that the activity of pyramidal cells is modulated by several classes of inhibitory neurons [26], [27], and that the interaction of excitation and inhibition induces prominent oscillations in multiple frequency bands [26], [28]. Yet, it is largely unclear whether and how these dynamical motifs contribute to efficient memory recall.

Here, we develop a theory that specifically addresses the problem of memory recall from synapses with a limited dynamic range, and thus consider how various neuronal and synaptic biophysical properties of area CA3 contribute to this process. We start by assuming that synaptic efficacies are limited and adopt one particular, oft-studied model of metaplasticity, the cascade model, where synapses make transitions between different states which have the same overt efficacy but differ in their propensity to exhibit further plasticity [17]. In order to understand how memories can be recalled efficiently from such synapses, we derive recurrent network dynamics that are optimal for this purpose. Our approach is based on treating memory recall as a probabilistic inference problem, in which the memory pattern to be recalled needs to be inferred from partial and noisy information in the recall cue and the synaptic weights of the network, and network dynamics act to produce activity patterns that are representative of the resulting posterior distribution. Given the statistical properties of the prior distribution of patterns, the recall cues, and the learning rule, the network dynamics that we derive to be optimal for retrieval are fully specified without free parameters to tune (except, as we show later, for some parameters affecting the speed of recall). The essence of our approach is that there is a tight coupling between the specifics of the learning rule governing memory storage and the dynamics of the circuit during recall. This approach has already helped reveal some basic principles of efficient memory recall in neural circuits [14], [29]–[31], but has not yet been applied to bounded metaplastic synapses.

While we derived optimal recall dynamics with only minimal *a priori* regard to biological constraints, we found that approximately optimal retrieval can be achieved in a neural circuit whose basic functional structure resembles the standard, biophysically motivated dynamics used for additive learning rules [4]. Importantly, the solution involves several critical motifs that are not predicted by standard approaches, and yet map onto known features of the dynamical organisation of hippocampal area CA3. First, precisely balanced feed-back inhibition [32] and pre- and postsynaptic forms of intrinsic plasticity (IP) [25], [33] matched to the form of synaptic plasticity that stores the memory traces, are necessary for ensuring stability during retrieval. Second, oscillations that periodically change the relative contributions of afferent and recurrent synapses to circuit dynamics [34], [35] can further improve recall performance by helping the network explore representative activity patterns more effectively.

In sum, addressing the computational challenges associated with effective retrieval of information from bounded synapses provides novel insights into the dynamics of the hippocampal circuitry implementing this function. Thus, our work extends previous approaches that sought to understand the basic anatomical and physiological organisation of the hippocampus [2], [36] as functional adaptions towards memory recall by providing a similar functional account of further crucial aspects of hippocampal organisation, involving plasticity and circuit dynamics.

## Results

We start by providing a formal description of autoassociative memory recall as a probabilistic inference task. We then derive recall dynamics that solve this task (approximately) optimally and investigate their computational and biological implications. First, we show that efficient recall is possible from metaplastic synapses with such dynamics. Second, as several details of the derived dynamics are unrealistic, we investigate biologically plausible approximations for them which enable us to identify the circuit motifs that are critical for effective memory retrieval in hippocampal circuits. Finally, we consider one particular improvement of the original solution which makes the recall dynamics more efficient and suggests a novel computational role for network oscillations.

### A probabilistic framework for autoassociative memory recall

**Figure 1:**
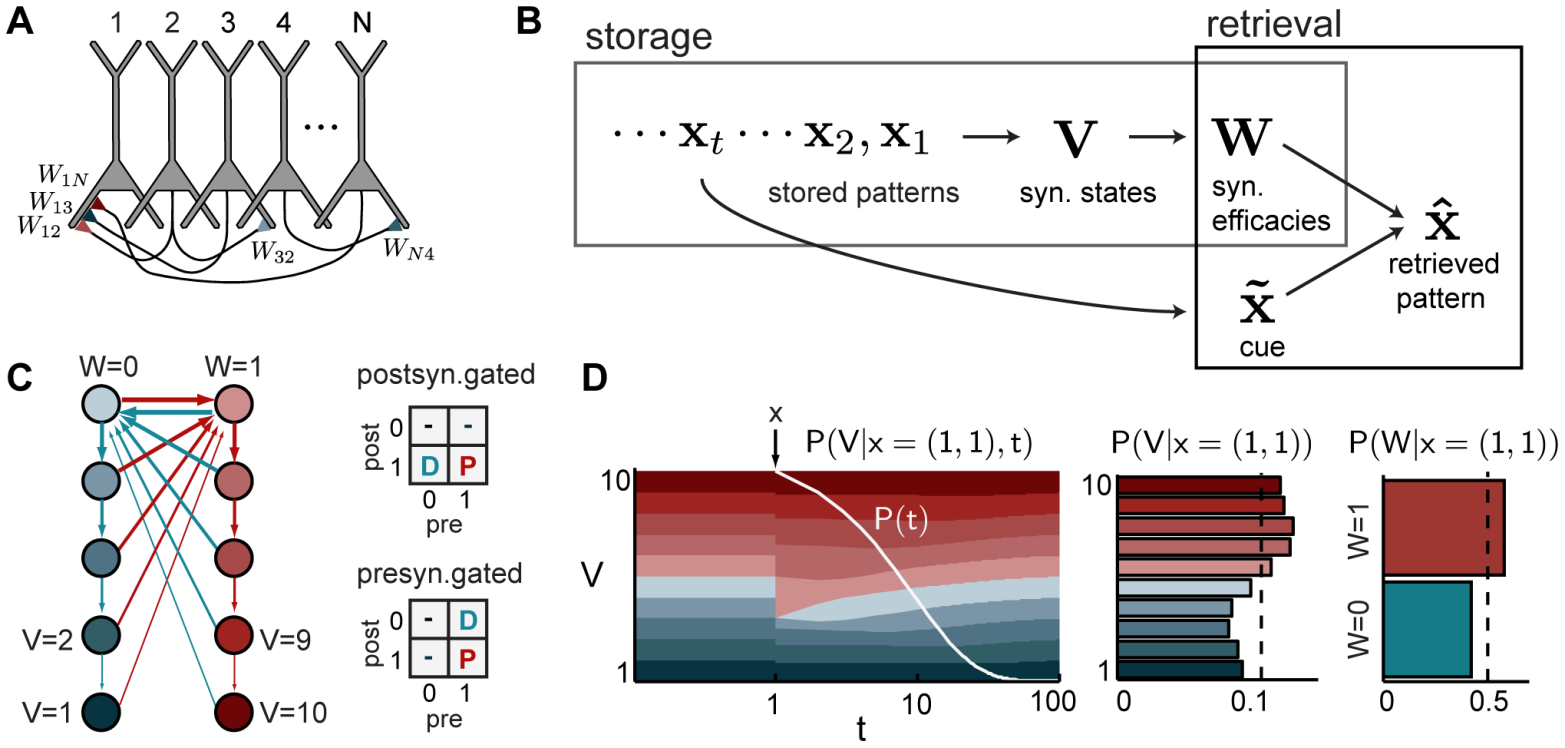
Autoassociative memory with bounded synapses. **A.** Memories are stored in the recurrent collaterals of a neural network. Five example synapses are shown, each in a different state (colors from panel C). **B.** During storage, a sequence of items, 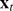 (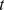 indexes time backwards from the time of recall), induces changes to the internal states, 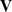, and thus to the overt efficacies, 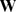, of recurrent synapses in the network. During retrieval, the dynamics of the network should identify the pattern to be recalled given a cue and information in the synaptic efficacies. **C.** The cascade model of synaptic metaplasticity [17]. Colored circles are latent states, 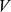, that correspond to two different synaptic efficacies, 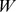; arrows are state transitions (blue: depression, red: potentiation). Tables show different variants of mapping pre- and post-synaptic activations to depression (D) and potentiation (P) under the pre- and postsynaptically-gated learning rules. **D.** Left: the evolution of the expected distribution over synaptic states (thickness of stripes is proportional to the probability of the corresponding state, see panel C for color code) after a potentiation event at time 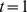 (marked by the vertical arrow) and the storage of random patterns in subsequent steps, and the distribution of times at which this memory may need to be recalled (white curve). Middle: the time-averaged expected distribution of hidden synaptic states at the unknown time of recall of this memory. Right: the corresponding distribution over overt synaptic efficacies.

We consider an auto-associative memory task in which a sequence of patterns, 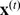, is stored by one-shot learning in the synaptic efficacies (or weights), 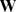, of the recurrent collaterals of a neural network. This models the network of pyramidal neurons in hippocampal area CA3. (We do not model other cell types or hippocampal subfields explicitly, but do consider their effects on CA3 pyramids, see also below). Here, 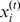 is the activity of neuron 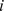 in the pattern that was stored 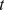 time steps prior to recall (in other words, the age of this pattern is 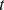), and 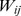 is the (overt) efficacy of the synapse between presynaptic cell 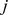 and postsynaptic cell 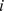 at the time of recall (Fig. 1A–B). For tractability, we assume that neural activities are binary; although extensions of the theory to analogue activities are also possible [14], [31].

#### Memory storage in cascade-type metaplastic synapses

Although we assume that the efficacy of a synapse, 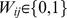, is binary, underlying these two ‘overt’ states there is a larger number of ‘hidden’ states, 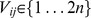, between which the synapse can transition, engendering a form of metaplasticity [21] (Fig. 1B–C). More specifically, we use a model in which synaptic plasticity is stochastic and local, with (meta)plasticity events inducing changes in the hidden state of each synapse, 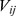, as a function of the activity of the pre- and postsynaptic neuron, 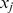 and 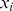 respectively [17] (see Methods for details). Each of these hidden synaptic states is mapped into one of the two overt binary synaptic efficacies, 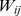, which can be used to influence network dynamics at recall.

We considered two possible rules for mapping the activity of the pre- and postsynaptic neuron into plasticity events: a postsynaptically-gated learning rule, with plasticity occurring whenever the postsynaptic neuron is active, leading to either potentiation when the presynaptic neuron is also active or to depression otherwise; and a presynaptically-gated learning rule, in which synaptic change occurs only if the presynaptic neuron is active (Fig. 1C). The first form seems more biologically relevant, as plasticity in hippocampal area CA3 is NMDA-receptor dependent (and hence requires postsynaptic depolarization for induction) [37], while the presynaptically-gated form has been traditionally assumed in past analyses of recall performance for autoassociative memory tasks [22], [38]. We followed common practice in setting the parameters of the model that determine the particular transition probabilities (see Methods), but did not otherwise attempt to set them explicitly to maximize information storage [39].

#### Memory recall as probabilistic inference

At the time of retrieval, the network is presented with a cue, 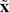, which is a noisy or partial version of one of the originally stored patterns, 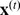 (Fig. 1B). Network dynamics should lead to a recalled pattern 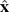 by combining the information in the cue, 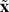, and the weights, 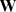 (Fig. 1B). Note that each of these sources of information alone is unreliable: the cue is is imperfect by definition (otherwise there would be no computational task to solve, as the cue would already be identical to the pattern that needs to be recalled), and the weights provide only partial information, because the synaptic plasticity rule is stochastic and the information about any particular memory pattern interferes with the effects of storing other patterns in the same set of synaptic weights (Fig. 1D).

Combining information from multiple unreliable sources, such as the recall cue and the synaptic efficacies, is inherently a probabilistic inference problem [29], [30]. In order to understand better what this problem implies, and to start our investigation of potential solutions to it, we first focus on the posterior distribution over patterns, which expresses the probability that pattern 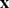 is the correct pattern to be recalled given the information in the recall cue and the weights:
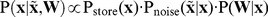
(1)where 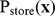 is the prior distribution from which patterns are sampled at the time of storage, 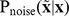 is the distribution describing noise corrupting the recall cue, and 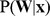 is the probability that the synaptic weight matrix is 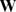 at the time of recall given that pattern 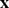 was stored some time in the past with a known synaptic plasticity rule (such as the one described above). Thus, in general, there are several patterns that may constitute the correct answer to a recall query, each with a different probability given by the posterior distribution, 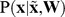 (Eq. 1). We consider a neural circuit to perform well in autoassociative recall if its dynamics are such that the resulting activity patterns are somehow representative of this distribution. However, before we spell out in detail the link between the posterior and actual neural dynamics (see next section), we first need to understand more thoroughly some key properties of the posterior, and in particular how it is affected – through the likelihood term 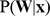 – by the synaptic plasticity rule used to store memories.

Previous analyses of optimal recall considered forms of synaptic plasticity that are mathematically unstable and biologically unrealistic. In these, unlike actual neural circuits [40], [41], synaptic weights do not have a proper stationary distribution. The most common case involves additive learning rules [4], [14], [31]. These make synaptic weights grow without bound and imply that the information available about a pattern is independent of pattern age. They thus do not correctly capture behavioural forgetting [42]. Conversely, storage in binary synapses with a logical OR-like rule [30] creates a degenerate stationary distribution for synaptic weights, because all synapses eventually become potentiated. It also makes forgetting catastrophically fast [17]. The cascade learning rule we investigate here covers the biologically relevant scenario in which synaptic weights have a well-defined, non-singular, stationary distribution (Fig. 1D). In this case, old memories are overwritten by the storage of new ones, but metaplasticity helps to maintain memories efficiently over long retention intervals [17].

Fig. 1D provides intuition for the four steps involved in computing 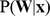 when synapses evolve according to the cascade model (for formal details, see Methods).

1. The evolution of the hidden synaptic states, 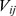, after storing pattern 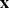, can be described by a stochastic process (formally a Markov chain), characterizing the probability of the synapse being in any possible state 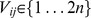 after storing a specific pattern 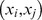 (the example shown in Fig. 1D is for 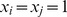), and then a set of subsequent patterns.
  There are three key stages in the evolution of the synaptic state. First, before storing 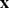, the state of the synapse reflects the large number of patterns that preceded it and were drawn from 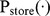. These leave the synapse in a stationary distribution which, in our case, is uniform. Thus in Fig. 1D (left) the thickness of the stripes showing the probability before storage, 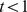 (with slightly informal notation), is the same for all possible synaptic states. (Note that 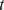 can equivalently denote the age of the pattern that needs to be recalled at any particular time, or the time elapsed since the storage of a particular pattern, starting with 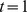 when the pattern is the last pattern that has been stored.)
  Second, at the time of storage, 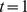, pattern 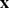 is stored in the synapse. In the particular example shown in Fig. 1D, both the post- and pre-synaptic cells are active in this pattern, i.e. 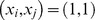. This triggers a potentiation event in the form of a stochastic transition between the latent synaptic states (following the red arrows in Fig. 1C). In this case, this increases the probability of the synapse being in states 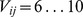, corresponding to 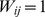.
  Finally, subsequent patterns stored after 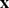, again drawn from 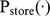, lead to similar stochastic transitions, ultimately determining the state of the synapses at the time of recall, 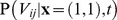. Formally, the effect of these other patterns can be described by repeatedly applying a single transition operator that averages over the possible identities of the other patterns (see Methods for details). From the perspective of the original pattern we aim to retrieve, all these subsequent patterns act as a source of noise, because they reduce the amount of information available in the synapses about the original pattern (the distribution becomes increasingly similar to that before storing the pattern, Figure 1D, left).
2. As the distribution over synaptic states at the time of recall depends on the (unknown) pattern age (i.e. the number of times the average transition operator has been applied since storing the original pattern), we need to integrate over the distribution of possible pattern ages 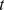, 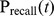 (Fig. 1D, left, white curve). Thus, we compute 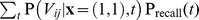, yielding the time-averaged expected synaptic state distribution, 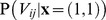 (Fig. 1D, middle).
3. As it is only the overt synaptic efficacies, 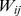, and not the hidden states, 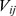, that can influence the interactions between neurons during recall, we apply the deterministic mapping between hidden synaptic states and overt synaptic efficacies to determine the probability distribution over the latter, 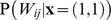, obtained by summing together the probabilities of 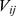 values that correspond to the same 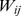 value (Fig. 1D, right).
4. Finally, in order to simplify our analysis, we assume that for local synaptic plasticity rules, for which the change in 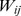 only depends on 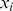 and 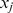, the evidence from the synaptic efficacies can be factorized as 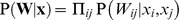 [14], [30] (but see [43]).

#### Dynamics for approximately optimal recall

As we saw above, the answer to a recall query lies in the posterior distribution, 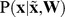 (Eq. 1). How can neural dynamics compute and represent it, even if approximately? While there exist several proposals for representing probability distributions in neural populations [44], [45], sampling-based methods offer a particularly suitable representational scheme. In this, each neuron corresponds to one random variable (one element of 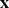), and thus the pattern of activities in the whole population at any particular time (the momentary ‘population vector’ [46]) represents one possible setting of the whole vector 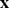. The key step is to show that biologically plausible interactions between neurons can lead to stochastic network dynamics (also known as Markov chain Monte Carlo [47]) which, over time, visit any particular state 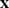 with just the right frequency, i.e., proportional to its probability under the posterior 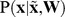 (for an illustration, see the stochastic trajectory trace in Fig. S1). Thus, the resulting sequence of activity patterns can be interpreted as successive samples taken from this high-dimensional posterior.

This representational scheme has the advantage that it is naturally suited to work when the number of variables over which a probability distribution needs to be represented is the same as the number of neurons in the system, as is conventional for associative memories. Furthermore, a sampling-based representation is also computationally appealing, as it allows the ‘best’ estimate (in the squared error sense) to be read off by simple temporal averaging, and also allows the uncertainty associated with this estimate to be characterized naturally by the variability of responses (Fig. S2). This uncertainty can then feed into higher order processes monitoring and modulating memory retrieval [48], [49].

While we discuss later some direct evidence for sampling-based representations of the posterior in the hippocampus [46], [50], [51], we have also considered several alternative neural representations of the posterior. These include representing the most probable pattern (maximum a posteriori, or MAP, estimate) [14], [30], or representing explicitly the (real-valued) probability of each neuron in the (binary) stored pattern being active (mean-field solution) [30], [31]. These approximations can be achieved by deterministic, attractor-like, dynamics, rather than the stochastic dynamics required by a sampling-based representation; nevertheless, we showed that the same circuit motifs arise in these cases as for the sampling case discussed below (see Text S1 and Fig. S1).

**Figure 2:**
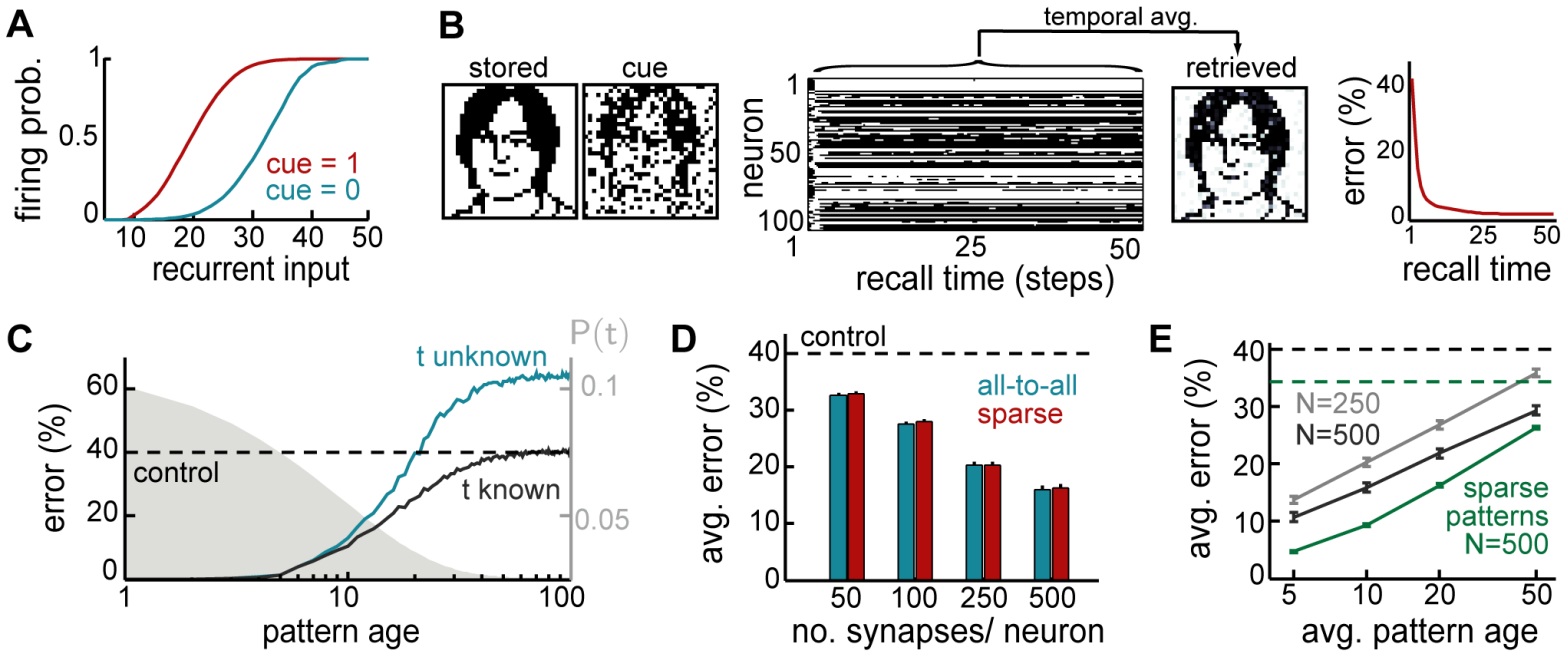
Optimal recall. **A.** Optimal neural transfer function: the total somatic current combines the recurrent contribution and a persistent external input corresponding to the recall cue. **B.** An example retrieval trial, from left to right: the pattern to be retrieved; the recall cue; activity of a subset of neurons during retrieval; final answer to the retrieval query obtained by temporally averaging the activity of the population; evolution of r.m.s. retrieval error over time in a trial. **C.** Recall performance as a function of pattern age (blue). As a reference, performance when the age of the pattern is known to the network is also shown (black, see Text S3). Gray filled curve shows distribution of retrieval times. **D.** Average performance as a function of the number of synapses per neuron in fully connected networks of different sizes (blue), or a sparsely connected network of fixed size 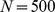 varying the number of connections (red). **E.** Average performance as a function of average pattern age in fully-connected networks of different sizes for balanced patterns (coding level = 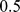, black, gray), and sparse patterns (coding level = 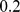, green). Dashed lines in panels C–E show, as a control, the performance of an optimised feed-forward network without synaptic plasticity (see main text for why this is a relevant upper bound on average recall error).

The particular form of sampling dynamics we consider is called Gibbs sampling [52], which, in general, requires the activity of each neuron 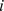 to be updated asynchronously by computing the probability of it being active conditioned on the current states of all other neurons (a vector denoted by 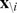). Formally, in each update step the activity of a randomly selected neuron 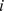 is computed by sampling the probability 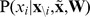. In our case, this is equivalent to the firing of a neuron being driven by a sigmoid transfer function (Fig. 2A):
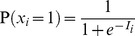
(2)with the total somatic current to the neuron, 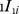, given as the log-odds ratio:
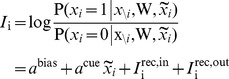
(3)and the contribution of the recurrent weights themselves given by:
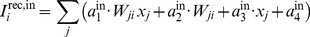
(4)

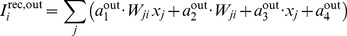
(5)The expressions for the current in equations 3–5 show the important result that the optimal way for a neuron to integrate inputs in its total somatic current is via a simple sum of neuron-specific terms (Eq. 3): a constant bias, 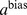, an input current from the recall cue, 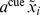, and terms that account for recurrent interactions within the network, 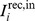 and 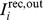, which themselves can also be expressed in a simple linear form, as a sum over the synaptic partners of the neuron (Eqs. 4–5).

Note that the general functional form of our dynamics resembles a stochastic version of a canonical model of recurrent network dynamics for autoassociative recall: the Hopfield network [53]. However, along with their broad conceptual similarity, there are also several distinctive features of our model that set it apart from Hopfield-like network models. First, the exact expression of the optimal current includes several terms that are not included in standard network models, but which will prove to be critical for efficient recall (Text S2). In turn, the same terms also correspond to biological processes not accounted for by previous models of autoassociative memory. Moreover, while making Hopfield-like neural dynamics work for the kind of realistic learning rules we are studying is very difficult, and at the very least requires considerable fine tuning of parameters [22], the parameters of our recall dynamics, 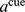, 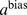, and 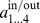, are all uniquely determined by the parameters defining the input noise and the storage process (i.e. the learning rule, the statistics of the stored patterns, and the pattern age distribution, see Methods).

By construction, the dynamics defined by equations 2–5 are optimal, in the sense that they will (asymptotically) produce samples from the correct posterior distribution. But are these dynamics neurally plausible? While our dynamical equations may seem somewhat abstract, previous work has shown that a network of simple stochastic integrate-and-fire-like spiking neurons, in which each neuron receives a total somatic current that is determined by the corresponding log-odds ratio (i.e. just as in our case, see Eq. 3), naturally implements precisely the same sampling procedure as our simpler Gibbs dynamics [54].

Hence, as long as the total somatic current has a realistic form, the complete form of the network dynamics can also be rendered realistic. For example, although the expression for the total current is semi-local, in that it depends only on the activity of the neuron's pre- and post-synaptic partners, it assumes an unrealistic symmetry in a neuron's ability to process information through its incoming and outgoing synapses (Eq. 4–5). Therefore, to address this issue, along with the biological implications of other features of our model, in the following we will focus on analysing properties of the total somatic current.

### Computational efficiency of approximately optimal recall

Having derived the approximately optimal dynamics for memory recall, we first study its efficiency by numerical simulations, using the simpler Gibbs dynamics (in light of their formal equivalence to a network of stochastic spiking neurons, see above). Specifically, our network dynamics proceed in discrete iterations corresponding to a full network update. In each such iteration, we first sample a random permutation to determine the order in which the neurons are to be updated, and then we update each neuron by applying Eqs. 2–5.

We first consider an example in which we store a specific pattern 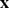, followed by a sequence of 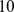 random other patterns (Fig. 2B, left). The retrieval cue, a noisy version of the original pattern, is used both as an initial condition at the beginning of recall and, as required by Eq. 3, also as a source of external input biasing the network throughout the retrieval process. The activity of the network is stochastic, asymptotically sampling the corresponding posterior distribution, and the output of the network, 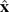, is taken to be the running temporal average of the network activity (Fig. 2B, middle). We measure retrieval performance by root-mean-squared (r.m.s.) error, which implies that the optimal response is exactly the posterior mean 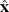. Even though sampling-based dynamics may, in general, suffer from slow convergence and mixing, as we will also show below, the particular dynamics here attains its asymptotic performance in only a few time steps (Fig. 2B, right). A useful corollary of these dynamics is that the variability of the responses during recall also represents a computationally relevant quantity: the confidence in the correctness of the output (the average activity). Indeed, as expected from a system with a well-calibrated representation of confidence, variability correlates strongly with the actual errors made by the network (see Fig. S2).

To evaluate the overall retrieval performance of the network more systematically, we repeat the storage and retrieval procedure described above 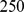 times. The patterns are drawn randomly from 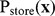, a uniform distribution over binary vectors, and the age of each pattern to be recalled is drawn from 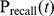, the prior over 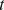 (Fig. 1D, left, white curve). For each pattern, we simulate the effects of storing 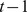 other random patterns on the synaptic weights, and then run our network dynamics, by starting it from the recall cue. At the end of each recall trial, lasting 100 time steps, we measure the error (normalised Euclidean distance) between the originally stored and the recalled pattern and average the errors across all trials.

We compare the average performance of the optimal network to that of a ‘control’ network which is a feed-forward network that retains no information about the particular patterns that have been stored, but does perform optimal inference given the general distribution of patterns and the recall cue (first two terms in Eq. 1). This should provide an upper bound on recall errors because it simply ignores the information in the recurrent collaterals. While this control may seem trivial, several classical recurrent autoassociative memory networks are, in fact, unable to outperform it [14], [43] (see also Fig. S3).

The performance of our recurrent network deteriorates as a function of pattern age (Fig. 2C, see also Text S3), as expected, but the average error across pattern ages reveals that the network performs significantly better than the control (Fig. 2D). In line with previous work that assumed additive synaptic plasticity [4], [14], retrieval performance is ultimately determined by the number of synapses per neuron (Fig. 2D). Due to the limited dynamic range of synapses, recall performance is also influenced by the average pattern age, such that a larger network (with more synapses per neuron) can recall older patterns more proficiently (Fig. 2E). A similar rescaling of errors is observed when using more biologically plausible sparse patterns [1] instead of the dense patterns we used in other simulations (Fig. 2E). In this case, the amount of information per pattern is reduced and so more patterns can be remembered. The quality of recall of the control (green dashed line) also improves, because the prior over patterns also becomes more informative (specifying *a priori* that most neurons should be inactive).

**Figure 3:**
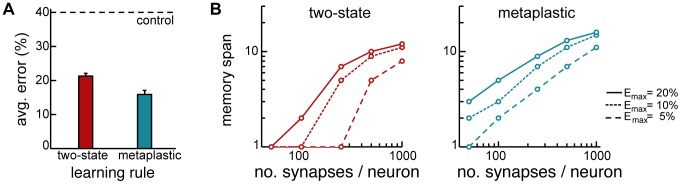
The advantage of metaplastic synapses. **A.** Recall performance in simple two-state (cascade depth 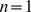) versus metaplastic (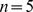) synapses. **B.** Scaling of memory span, defined as the maximum age for which patterns can be recalled reliably within the allowable error, 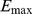, for two-state (left) and metaplastic (right) synapses.

Despite the well-known advantage of the cascade model over simple two-state synapses in storing information [17], [55], previous work using heuristically constructed recall dynamics was unable to demonstrate a similar advantage in recall performance [22]. Optimal dynamics confers substantial improvement in recall performance when synapses have multiple metaplastic states (Fig. 3A). Importantly, one of the hallmark benefits of metaplastic synapses is that the time for which they retain information after encoding scales as a power-law of the number of synapses per neuron, instead of the catastrophically poor logarithmic scaling exhibited by deterministic two-state synapses [17], [56]. The quality of information recall in our network shows the same scaling relationships, thus retaining this crucial advantage of metaplastic synapses (Fig. 3B).

Motivated by these findings, we now turn to the question of how these approximately optimal dynamics can be implemented, and further approximated, by neural circuit dynamics. For computational convenience, we will consider moderately sized all-to-all connected networks, dense patterns, and small average pattern ages, but these results generalise to the more realistic case of large sparsely-connected networks recalling sparse memories after longer retention intervals (as suggested by Fig. 2D–E). We will take special care to assess the effects of sparse connectivity in those cases in which the detailed structure of the connectivity matrix can be expected to matter for recall performance.

### Neural implementation of approximately optimal recall

The dynamics defined by equations 2–5 have two appealing properties. First, by construction, they represent an approximately optimal solution to autoassociative recall, with all the parameters of the recall dynamics being derived from those characterising memory storage and the input noise. Second, at the same time, they are in a form that is in a loose agreement with standard reduced models of single neuron dynamics (thresholded, linear summation of inputs). However, several details of the dynamics are unrealistic, and it is therefore necessary to show whether and how these details can be approximated by a neural circuit without severely compromising recall performance. Conversely, neuronal dynamics in cortical areas such as CA3, that may be involved in the recall of associative memories, exhibit features that are mysterious from the perspective of recall based on conventional, additive, plasticity rules. We consider the possibility that these features might play a role in the approximations.

A common theme in the approximations we are to consider is to replace an original, implausible term of the total somatic current by its statistical average. Averages can readily be taken over the activity of the population (as we will see when we consider the role of the balance between excitation and inhibition), or over the statistics of previous patterns (as we will see when we consider pre- and/or post-synaptic forms of intrinsic plasticity). In general we ask two questions about each approximation:

- Is it efficient, i.e. is recall performance close to that seen with the exact dynamics?
- Is it necessary, i.e. is it possible to achieve the same performance by an even simpler approximation?

The conclusion of the following sections will be that, in fact, several aspects of neural circuit organisation characterising hippocampal area CA3 can be understood as such necessary and efficient approximations.

It is important to note that we only explicitly model pyramidal neurons (the principal cells) in CA3, and that all other mechanisms involved in implementing approximately optimal memory recall will be described phenomenologically, in terms of their effects on the total somatic current of pyramidal neurons – which is the only computationally-relevant quantity in our model. Nevertheless, for each of these mechanisms, we will point out ways in which they may be dynamically implemented in the neural substrate and also quantify their effects in a way that allows direct comparisons with experimentally measurable quantities (see also Discussion).

#### Intrinsic plasticity

The most obviously unrealistic feature of the optimal recall dynamics derived above is that incoming and outgoing synapses to a neuron should both contribute directly to the total somatic current (Eq. 3). As synaptic transmission is unidirectional, the term corresponding to the outgoing synapses, 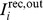, needs to be approximated. We investigated three different approximations of increasing complexity, all of which are based on (conditional) expectations of this quantity:
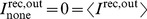
(6)

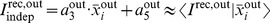
(7)

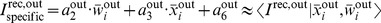
(8)where 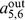 are constants defined by the statistics of the stored patterns and the learning rule (see Methods).

The simplest approximation, 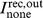, replaces 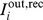 by its unconditional expectation [54], which in our case is 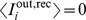 (Eq. 6). As it predicts no influence from outgoing synapses, we will refer to this approximation as ‘none’. The remaining two approximations are based on the conditional expectation of 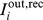, conditioning on sources of information that may be available to the neuron (see below). 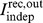 is conditioned on the summed activity of the neuron's postsynaptic partners, 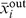, and as such it is still independent of the synaptic weights of the particular neuron (Eq. 7). The last and most sophisticated approximation, 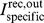, conditions on both 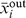 and on the sum of the outgoing synaptic weights of the specific neuron, 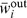 (Eq. 8). To minimise the complexity of the two more sophisticated conditional expectations, we further approximated them as sums of terms (plus a constant) that each depend linearly on one of the quantities on which they are conditioned (Eqs. 7–8).

There may thus be two quantities that need to be available to a neuron so that it can implement these approximations: 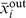 and 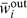. The magnitude of 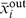 should vary over time within a recall trial, and we consider how it can be furnished by feedback inhibition in the next section. The magnitude of 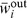 is constant on the time-scale of a recall trial, and acts as a bias term shifting the transfer function of the cell in the same way as the recall cue does in Fig. 2A. As we will show below, such a bias can be provided by a process which corresponds to a form of intrinsic plasticity that adjusts the excitability of the cell as a function of the strength of *outgoing* synapses, and to which we thus refer as 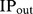. In order to assess the computational importance of these terms, and thus of the corresponding biological processes, we used numerical simulations as described above to compare the recall performance of three networks, each using one of the approximations in equations 6–8.

**Figure 4:**
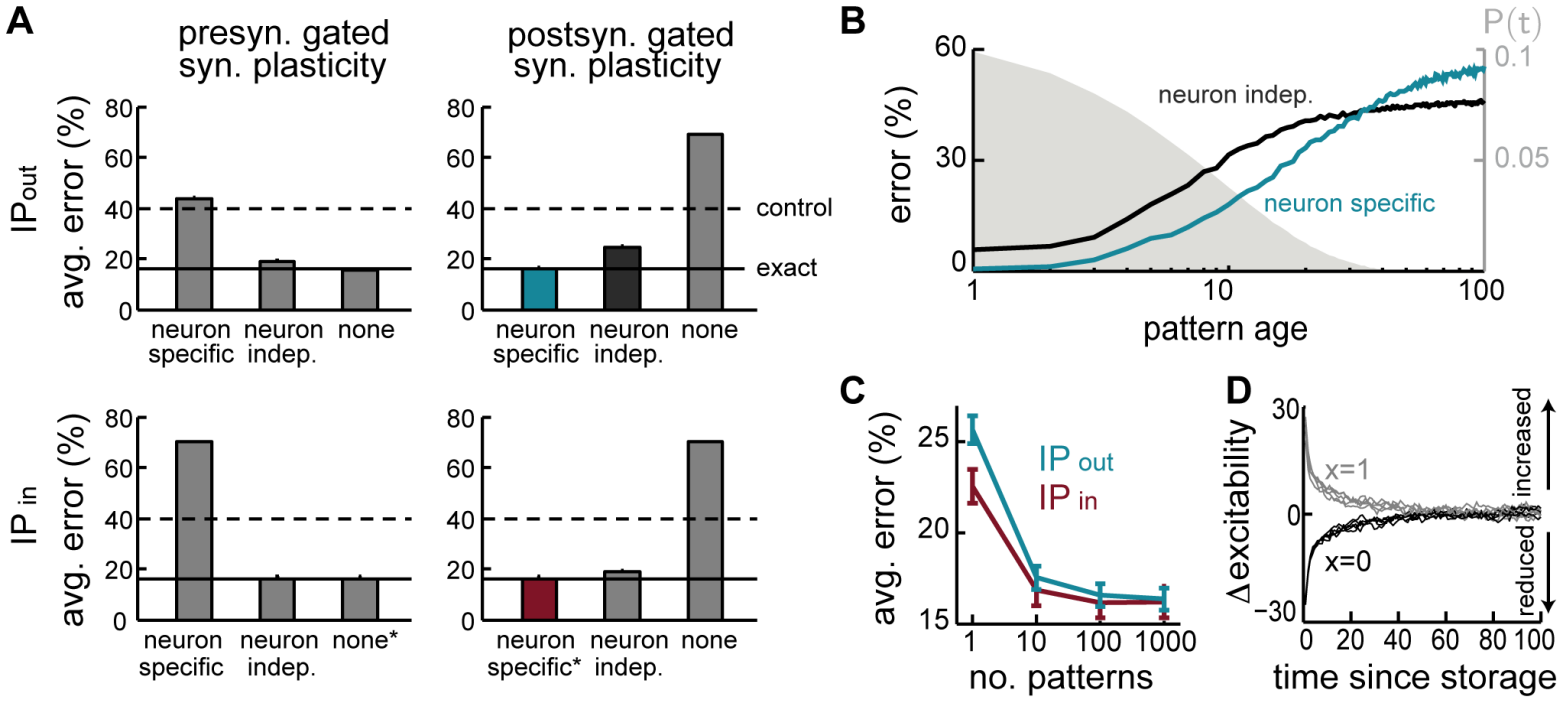
Intrinsic plasticity. **A.** Effects of different forms of IP (rows) for different forms of synaptic plasticity (columns). Recall performance is shown for different variants of each form of IP (bars), entailing different approximations of the exact (optimal) dynamics. Dashed lines show control performance of an optimized feedforward network, as in Fig. 2C–E; solid lines show performance of exact dynamics, asterisks mark neural dynamics that are formally equivalent to the exact case. **B.** Recall performance as a function of pattern age with neuron-independent (black) and -specific (blue) variants of 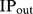 for the postsynaptically-gated learning rule. Gray filled curve shows distribution of pattern age. **C.** Recall performance for an online implementation of the two forms of IP. **D.** Net change in excitability induced by the two forms of IP together as a function of time since memory storage for neurons that were either active (gray) or inactive (black) in the originally stored pattern. Lines correspond to different random sequences of consecutively stored patterns.

Comparing the three approximations we have introduced above reveals an interesting dissociation between pre- and postsynaptically-gated synaptic plasticity rules (explained in Fig. 1C). The two rules behave identically when using the exact recall dynamics (as expected, because the synaptic weight matrix produced by them is identical up to a transpose operation). However, the effectiveness of the different approximations depends critically on the specifics of the synaptic plasticity used for encoding (Fig. 4A, top).

While the two simple solutions, 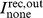 and 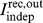, work well for the presynaptically-gated learning rule (Fig. 4A, top left), they fare considerably worse for postsynaptically-gated learning (Fig. 4A, top right). In the presence of intrinsic plasticity, network performance becomes very close to that achieved by the exact dynamics for the postsynaptically-gated rule (Fig. 4A, top right), but not for the presynaptically-gated learning rule (Fig. 4A, top left). This latter, slightly counterintuitive, effect is due to the linear approximation of the conditional expectation (Eq. 8), which ignores correlations between the synaptic efficacies of outgoing synapses and the activity of the postsynaptic neurons. The same linear approximation has no detrimental effect for the postsynaptically-gated rule. Moreover, the benefit of the weight-specific approximation for the postsynaptically-gated rule is particularly significant for recent patterns that could potentially be recalled well (Fig. 4B).

In sum, our theory predicts that the storage of memories by a hippocampal form of synaptic plasticity, which is postsynaptically-gated, should be accompanied by the appropriate form of IP for maintaining near-optimal performance. This IP is predicted to have a non-trivial form: it affects the presynaptic neuron (because it depends on the outgoing synapses), and it is ‘anti-homeostatic’ in that the potentiation or depression of the synapses between two cells should be accompanied by a respective increase or decrease in the excitability of the presynaptic cell. Interestingly, recent reports demonstrated a form of 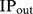 that followed precisely this pattern [57], [58].

The expression for the other main component of the total somatic current dictated by the optimal dynamics, 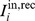 (Eq. 4), is also problematic biologically. Although computing 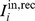 only requires information about the strength of incoming synaptic weights, there is a term in it that depends on the sum of these weights directly rather than the sum of currents (weights multiplied by presynaptic activities) through the incoming synapses. As we saw above in the case of outgoing synapses, sums over synaptic weights can be approximated by adjusting neural excitability, and hence this suggests that, beside 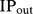, there should also be a postsynaptic form of IP that regulates a neuron's excitability depending on the sum of *incoming* synaptic weights, 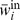, and to which we refer as 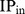.

Therefore, we again constructed three approximations to this term that were analogous to those used for outgoing synapses (Eqs. 6–8), but replaced 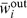 by 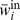. Interestingly, the need for 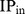 is specific to the postsynaptically-gated learning rule, as the constant factor multiplying 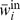, 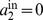 vanishes for the presynaptically-gated rule, see Methods. Unlike in the case of 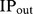, this term is homeostatic in nature (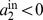 for the postsynaptically-gated rule), with neurons becoming less excitable when many of their incoming synapses are strong. Such homeostatic regulation of the postsynaptic neuron's excitability is well documented experimentally, and is believed to play an important role in modulating neuronal activity during learning to ensure network stability [25]. The same principle applies in our model, as removing this regulation has catastrophic consequences for retrieval, and even replacing this term with a neuron-independent form of homeostatic regulation still impairs network performance (Fig. 4A, bottom right). Moreover, this impairment becomes dramatically worse in sparsely connected network (not shown). Conversely, introducing a homeostatic regulation term in the case of the presynaptically-gated learning rule has equally detrimental effects (Fig. 4A, bottom left). This reinforces the notion that a tight match is needed between the form of the synaptic plasticity rule storing memories and the presence and form of mechanisms regulating neural excitability.

Although a direct dependence of neuronal excitability on the strength of net incoming and outgoing connections, as proposed above, may seem difficult to achieve biologically, it can be well approximated by a temporal average of the incoming (or outgoing) excitatory drive to the neuron, as it is commonly formalised in standard models of IP [59], [60]. Essentially, this requires estimating the average current into the neuron when the incoming inputs are distributed according to the prior over stored patterns (by averaging over responses during other retrieval trials, see Methods for details). The effectiveness of this approximation depends on the time scale for integrating past activity, and needs to be at least an order of magnitude slower than the time scale on which individual memories are stored and retrieved (Fig. 4C). Thus, this process makes the neural threshold neuron-specific and keeps it fixed on the time scale of individual recall trials, while slowly updating it to reflect the history of patterns stored in the network. This is consistent with experimental evidence suggesting intrinsic plasticity be a slow process relative to the induction of synaptic plasticity [25].

While postsynaptically-gated plasticity consistently predicts the need for mechanisms regulating neuronal excitability, it is not immediately clear what the net effects of the two distinct forms of IP, 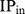 and 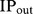, should be. In fact, at first glance, they seem to have opposite effects on neural excitability, with 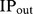 acting in a positive feedback loop, with neurons having a strong contribution to the drive of their postsynaptic partners becoming more excitable, and 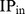 acting homeostatically, reducing neural excitability for neurons receiving many strong inputs. Predicting the net effect of the two processes is further complicated by the asymmetry in the learning rule itself, which makes it nontrivial to determine the changes in net synaptic strength into and out of a neuron. To investigate this question directly in a way that allows experimentally testable predictions, we monitored the changes in excitability in individual neurons triggered by storing a specific pattern, and the evolution of these changes with pattern age (Fig. 4D). We found that neuronal changes in excitability are ultimately dominated by the positive feedback process, with neurons activated or deactivated in the original storage event displaying an increase or decrease in excitability, respectively. This effect is general, and does not depend on the details of the synaptic plasticity rule as long as it is postsynaptically-gated. Furthermore, this effect is predicted to decrease with pattern age, following the time constant of synaptic forgetting.

#### Dynamic feedback inhibition

Another important consequence of the optimal retrieval dynamics derived above is that the total current to a neuron should include a negative contribution proportional to the population activity of its pre- and possibly postsynaptic partners. While earlier theoretical work already considered the importance of inhibition during retrieval [36], and several standard models of spike-based recurrent circuits exhibit a linear dependence of inhibition on the level of excitation [61], [62], our model advances these findings by predicting a specific form of feed-back inhibition that is both temporally and spatially specific. Temporal specificity requires that inhibition be dynamically regulated to match the level of excitation in the network. Spatial specificity requires that the level of inhibition received by each neuron should be determined by just the right pool of excitatory neurons (i.e., those ones with which it is connected). We investigated the importance of both forms of inhibitory specificity.

**Figure 5:**
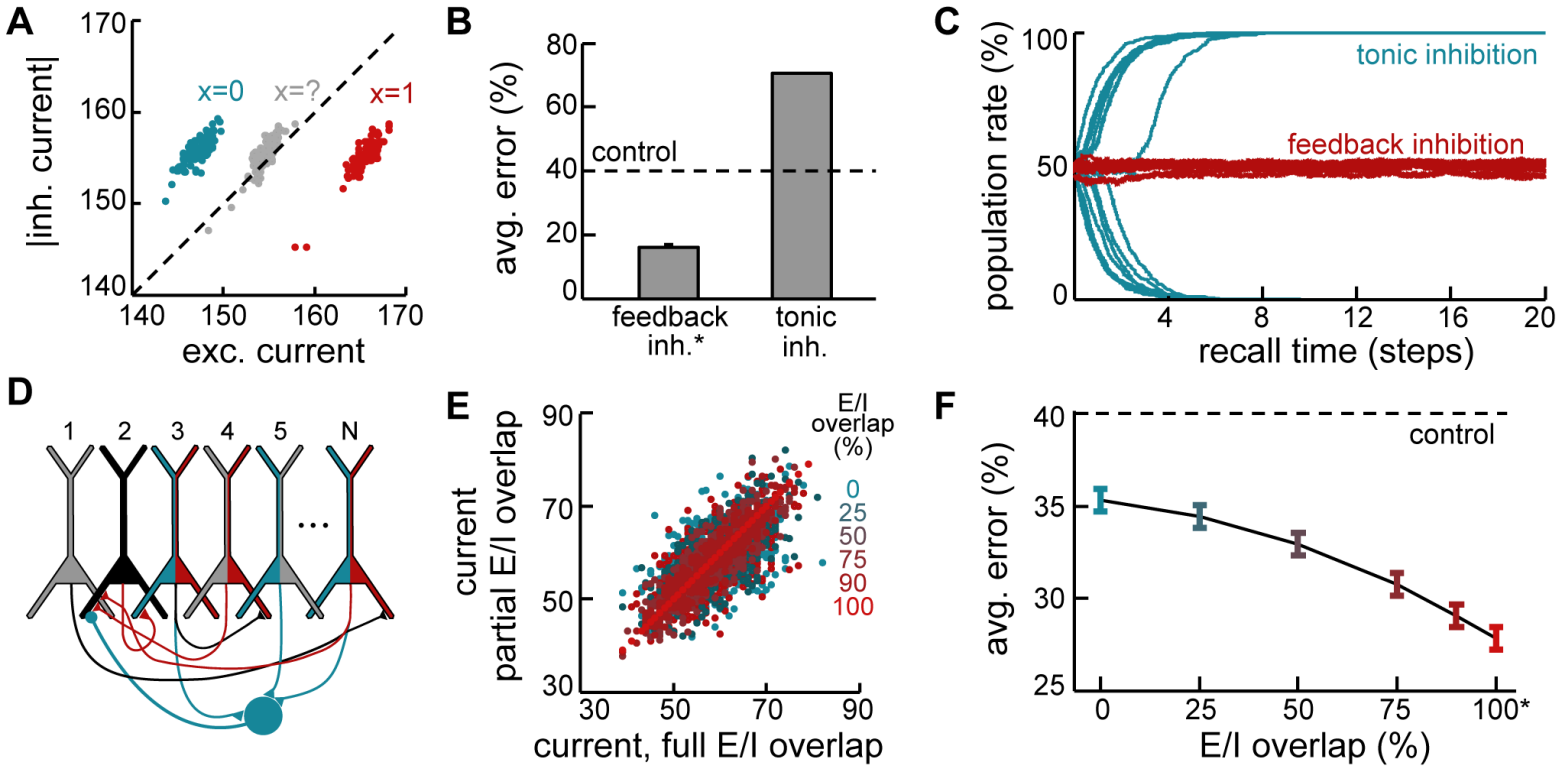
Dynamic feedback inhibition. **A.** Example statistics of inhibitory vs. excitatory currents to three example neurons during a recall trial. Blue: neuron correctly recalling a 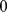 bit in the originally stored pattern, correctly recalled; red: neuron correctly recalling a 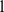 bit in the originally stored pattern; gray: neuron with high variability during the trial, corresponding to an incorrectly recalled bit. Individual dots correspond to different time steps within the same recall trial. **B.** Effect of replacing feedback inhibition by tonic inhibition with the same average level. **C.** Evolution of the mean population activity during retrieval when network dynamics involve feedback (red) versus tonic inhibition (blue). Lines correspond to different trials. **D.** Schematic view of inhibitory connectivity in the network. Pyramidal neurons sending or receiving monosynaptic excitation (disynaptic inhibition) to example neuron 2 (black) are colored red (blue). Blue circle: local interneuron (not explicitly modeled) mediating disynaptic lateral inhibition received by neuron 2. E/I overlap is measured as the ratio of presynaptic pyramidal neurons colored both blue and red, 0% is chance. **E.** Total somatic current (through recurrents) to an example cell in a sparsely connected network (20% connectivity) with full (x-axis) or partial E/I overlap (y-axis, colors); different points correspond to different time steps. **F.** Recall performance as a function of E/I overlap. Asterisks in B and F indicate network configurations that are formally equivalent to the exact dynamics.

Temporal specificity in the model leads to inhibition closely tracking excitation in single neurons, with the difference in magnitude between the two reflecting the evidence in favour of the neuron having been active in the pattern to be retrieved (Fig. 5A). In fact, the stabilisation of neural dynamics during retrieval relies heavily on such dynamically balanced feedback inhibition. Replacing the corresponding term in the total current term by its average value, which corresponds to replacing feedback by tonic inhibition, has catastrophic consequences for retrieval performance (Fig. 5B), as network activity becomes unstable, and – depending on pattern age and initial conditions – either explodes or dies out altogether (Fig. 5C).

Spatial specificity in the model requires a precise overlap for each neuron (for example, black neuron in Fig. 5D) between the population of those excitatory cells that are pre- or postsynaptic to the neuron (Fig. 5D, neurons with red fill) and the population that provides disynaptic inhibition to it (Fig. 5D, neurons with blue fill). While this can be trivially guaranteed in fully connected networks, it requires considerable fine tuning in realistic, sparsely connected networks. Although inhibitory plasticity has been suggested to tune inhibitory inputs to match excitation [63], it remains an open question how precisely biologically realistic synaptic plasticity of inhibitory circuits can realise such a match. Thus, we investigated the robustness of the recall dynamics to perturbations of the optimal inhibitory connectivity by systematically varying the probability that an existing source or target of monosynaptic excitation is also a source of disynaptic inhibition while keeping the total inhibitory input to each neuron constant (see Methods).

Perturbing the precise pattern of inhibition needed for optimal recall acts as a source of noise in the total current to the neuron (Fig. 5E). This depends on the excitatory/inhibitory (E/I) overlap, which is 0% for random connectivity, 100% for a precise match. This noise translates into an impairment in retrieval performance which also varies with E/I overlap, with the network continuing to perform significantly better than control even at small degrees of overlap (Fig. 5F). (In these simulations, the lower bound on achievable error, given by the exact recall dynamics, is relatively high due to the reduction in the number of synapses per neuron in the sparsely connected network.) Importantly, although some information is lost due to this approximation, the dynamics remain stable to perturbations in inhibitory connectivity, suggesting that approximately optimal dynamics could be realistically implemented in sparsely connected neural circuits without an exquisitly fine tuning of inhibitory connections.

#### The cumulative effects of biological approximations

**Figure 6:**
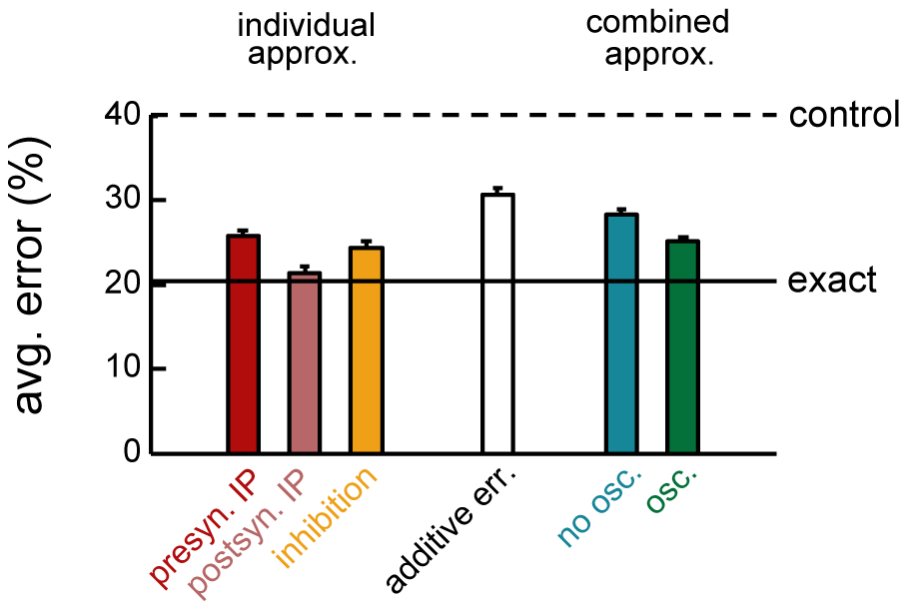
Combining different circuit motifs for approximately optimal retrieval. Retrieval performance with individual approximations (left), and all approximations combined (right), compared with a hypothetical scenario cumulating errors additively (middle). All networks are 50% sparsely connected. Dashed and solid lines show performance of exact dynamics and control network. Approximations used: online neuron-specific pre- (red) and postsynaptic IP (pink) with an online integration window of 10 patterns, 50% E/I overlap (yellow), all combined (blue), with additional population oscillations (green, see also Fig. 7).

Although we have shown that individual terms of the optimal recall dynamics can be approximated via biologically plausible mechanisms with relatively small detriments in recall performance, it is unclear whether the network can still work appropriately with all these approximations in place. To test this, we constructed retrieval dynamics combining the online form of both pre- and postsynaptic IP, and assumed a 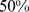 overlap between the excitatory and inhibitory input sources to neurons in the network (with 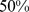 sparse network connectivity). We considered two sets of comparisons: to exact sampling dynamics, which provides a lower bound for the error rate achievable by our approximation, and to networks involving the various components individually (Fig. 6). We found that the performance of the biologically realistic network involving all approximations remained close to that of the optimal network. Indeed, the relative error of the whole set of approximations (compared to that of the exact sampling dynamics) was less than the sum of relative errors of the individual approximations.

### Population oscillations

Although Gibbs sampling was an attractive starting point for deriving dynamics that both work well in practice and can be related to biologically plausible neural network dynamics [54], it suffers from a major computational shortcoming: sloth. This means that a very large number of iterations may be necessary before the samples are appropriately distributed (i.e., a long burn-in time). Further, the alacrity with which Gibbs sampling explores this distribution may be limited (slow mixing). This would mean that consecutive samples are highly correlated, implying that very many of them would be needed to compute reliable expectations under the distribution [64], [65] thus compounding the error in the output of our network – which is computed as just such an expectation (Fig. 2B). These problems become particularly acute when the posterior distribution that needs to be sampled is multimodal (when modelling hippocampal flickering, see below) or itself exhibits strong correlations (e.g. corresponding to strong coupling in frustrated Ising models). In fact, similar problems affect the alternative, deterministic mean-field or MAP dynamics (discussed in Text S1) which suffer from local optima and regions of the objective function that gradient-based methods find hard to traverse.

#### Recalling old memories

**Figure 7:**
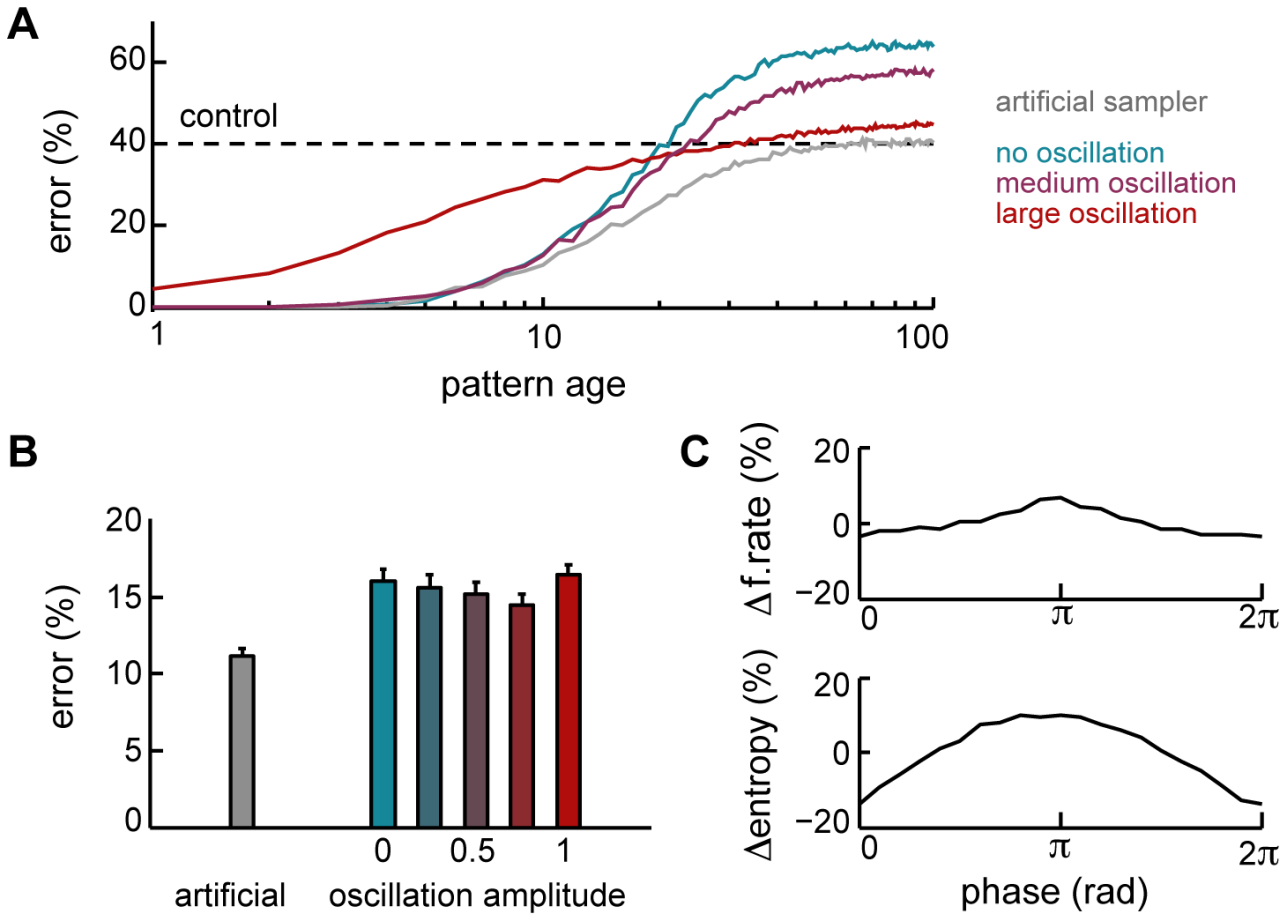
Population oscillations. **A.** Recall performance as a function of pattern age with optimal network dynamics without oscillations (blue, cf. Fig. 2C), with medium- (purple) or large-amplitude (red) oscillations, and with an artificial sampling algorithm (gray). **B.** Average recall performance with the artificial sampling algorithm (gray) and with different levels of amplitude modulation for network oscillations (amplitude 0.75 corresponds to the ‘medium oscillation’ in panel A). **C.** Average normalised population activity and response entropy at different phases during a cycle of a large-amplitude oscillation.

Fig. 7A (gray vs. blue) presents evidence for the infelicity of Gibbs sampling. It shows that a large fraction of the errors suffered by our network is solely due to slow convergence speed: an artificial sampler which samples the exact same posterior distribution but more efficiently [49] (see Methods) performs substantially better. The difference between our network and the artificial sampler is particularly striking for old memories: this is because for these, the entropy of the posterior distribution is large, and so a great number of different states needs to be visited for it to be represented fairly.

How can we improve retrieval dynamics in our neural dynamics to reduce the errors due to inefficient sampling? One potential solution, inspired by work in optimisation and sampling is to use annealing [64], [65]. In this procedure, the function that needs to be navigated – in our case the (log) posterior – is gradually ‘morphed’ between an approximate form, that is easy to handle, and its original form, that is hard, with the degree of morphing being controlled by a ‘temperature’ parameter. In the context of sampling in particular, an annealing-based procedure termed tempered transitions (TT) has been proposed as a way to ensure a more efficient exploration of the state space [64] (Fig. S4). This sampling procedure involves a form of oscillatory dynamics which periodically increases and decreases the ‘temperature’ parameter in way that could potentially be implemented by appropriate population oscillations in a neural circuit.

To construct TT-based dynamics for our problem, a naïve choice for the approximate distribution at the highest temperature would be a uniform distribution. However, while the uniform distribution is trivially easy to sample from, it is too unspecific as it retains no information about the original posterior and thus runs the risk of leading to inefficient sampling. Fortunately, there is a better option for the maximum temperature approximation: the combination of the prior over patterns and the likelihood for the recall cue. This approximation still retains important aspects of the posterior (the first two terms comprising it, see Eq. 1) while avoiding all the correlations in the posterior of which the sole source is the likelihood of the weights (the last term in Eq. 1). Therefore, it is a more efficient approximation than a uniform distribution, but it is equally easy to sample from (because it is fully factorised), and – as it is exactly the distribution sampled by the feed-forward network that we have used as a control – it is also readily implemented in the same recurrent circuit that represents the full posterior by simply suppressing the effects the recurrent connections.

As a result of using this more efficient approximation at high temperatures, the TT-based sampler results in network dynamics very similar to those corresponding to our original Gibbs sampler (Eqs. 3–5), with only two alterations. First, the relative contribution of recurrent inputs compared to that of external feed-forward inputs (corresponding to the recall cue) needs to be modulated in an oscillatory fashion (Methods). Such periodic modulation could potentially be implemented by a form of shunting inhibition differentially affecting distal and proximal synapses, corresponding to feed-forward and recurrent inputs, respectively [34], [35]. (As above, we do not explicitly model the inhibitory population which would provide this oscillatory input, only its effects on the total somatic current of the principal cells; there exist several spiking neuron models that could generate the required signal, see e.g. [66]). Second, the readout of population activity needs to occur at the phase of the oscillation corresponding to the original posterior (temperature = 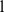). This could also be achieved by an appropriate oscillatory modulation of the Schäffer collaterals (the efferent fibers of CA3, see also Discussion).

One further approximation is required. In order to sample from the exact distribution, TT normally requires a step in which a possible sample taken at the lowest temperature after the whole oscillation, could be rejected as a whole. The state of all the neurons should then be returned to their original activities before the sample was created. This is a highly non-local operation in space and time, and so we made the approximation of omitting it.

Importantly, although the oscillatory dynamics we have introduced are only approximate, they are still helpful in speeding up convergence, allowing old memories to be retrieved substantially more competently (Fig. 7A, red vs. gray). Unfortunately, the same oscillatory dynamics prove to be detrimental when recalling recent memories. This is because the synaptic weights retain substantial information about these [17] implying that the posterior distribution is very concentrated, which is inconsistent with the over-exuberant changes in state that happen at the higher temperatures in TT. In the exact forms of this procedure, such moves are penalised by the highly concentrated posterior, leading to high rejection rates and slow dynamics. The approximate sampler, which lacks rejection, becomes less accurate. Therefore, there is an inherent trade-off in the utility of oscillations: the more useful they are for recalling remote memories, the more damaging they are for the recall of recent memories. Parametrically varying the amplitude of the oscillations reveals that an intermediate oscillatory strength, where the dynamics take into account recurrent inputs throughout the cycle (see Methods), best resolves this tradeoff (Fig. 7A, purple and Fig. 7B).

Generating network oscillations is almost unavoidable in a network combining excitatory and inhibitory neurons. However, the kind of oscillations we employ here have several characteristic signatures that can be used for experimentally validating our predictions. In particular, since the oscillation phase controls the temperature used to anneal the posterior distribution, the activity at the trough of the oscillation (phase 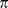, highest temperature) should correspond to samples from a broader distribution (Fig. S4). Hence, neural responses at the trough of the oscillation should be more variable than those at the peak. Indeed, if we measure the average entropy of the responses in the network as a function of the phase of the oscillation, response variability is predicted to be modulated with the period of the underlying oscillation, with most variability at the trough (Fig. 7C, bottom). An intriguing prediction that the overall level of population activity should be modulated much more weakly by the same oscillation (Fig. 7C, top). This is because, in the model, oscillations improve convergence speed by periodically modulating the spread of the distribution from which the network needs to sample (Fig. S4A), rather than by biasing it in any particular way, e.g. towards higher firing rates.

#### Representing spatial ambiguity

We have argued that oscillations help the network explore a broad posterior resulting from limited information in the synapses. Another particularly revealing regime involves multimodal posteriors. Such distributions might arise when animals receive conflicting cues, for instance, after an instantaneous change in spatial context. In this scenario, current sensory inputs suggest that the animal is in a new context, while the generally correct assumption that spatial contexts are contiguous in time suggests that the animal is still in the previous context, thereby creating substantial spatial ambiguity.

**Figure 8:**
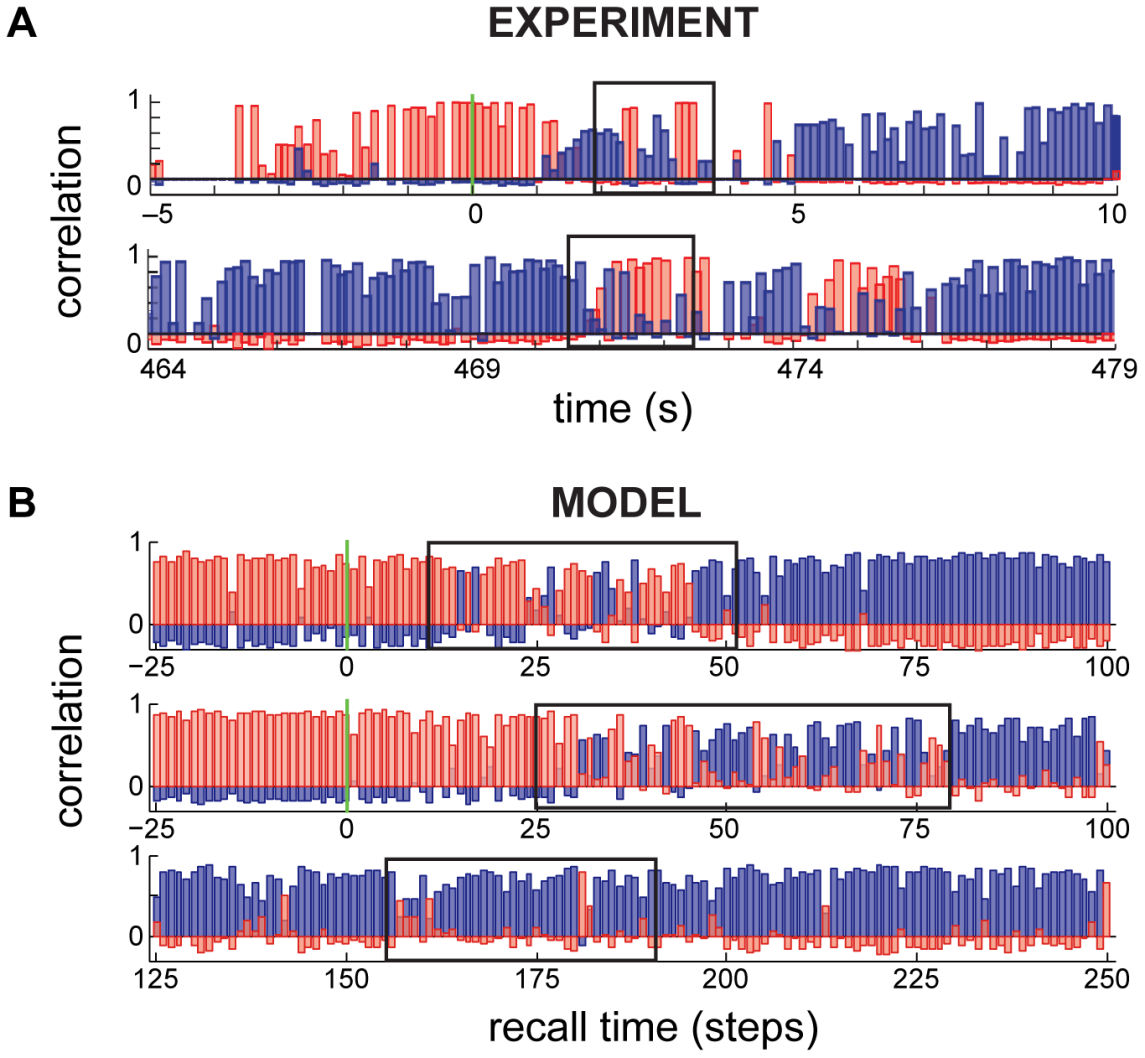
Network flickering. **A.** Hippocampal population dynamics during a single retrieval trial, reproduced from Ref. [46]. Correlation of the instantaneous population vector to the stereotypical responses of the network in the two contexts are shown (red vs. blue) Top: flickering (box) following the switching of visual cues at time 0 (green vertical line), bottom: spontaneous flickering (box) without external cue switching. **B.** Dynamics of population responses in the model showing flickering (boxes) after cue switching (top), and spontaneously, without cue switching (bottom).

The effects of spatial ambiguity have recently been examined in experiments recording place cells in rats experiencing just such rapid and abrupt changes between different spatial contexts [46], [50], [51]. Immediately following a switch in spatial context, and before hippocampal activity settled to representing the new context, transient flickering was observed, in which there was rapid switching back and forth between the recall states representing the previous and the new context (Fig. 8A; top), in a manner that was paced by oscillations in the theta range [46] (or, in a different experiment, the gamma range [51]).

When the effective recall cue is the recent history of sensory inputs (which is statistically appropriate since spatial location should only change slowly and smoothly under normal circumstances, see Methods), our network also generates transient flickering (Fig. 8B, top). In fact, as the net information available in the cue always remains limited, it never perfectly excludes other contexts, such that transient flickering can also be observed without switching, albeit much less frequently (Fig. 8B, bottom). Such spontaneous flickers were also observed in the original experiment (Fig. 8A, bottom; [46]).

## Discussion

A venerable history of classical theoretical work on hippocampal area CA3 accounted for many of the architectural and anatomical features of this area in terms of how they support its function as an autoassociative memory system [1], [2], [36], [67]. However, the *dynamical* behaviour of CA3 has so far escaped such a theoretical treatment. Indeed, unlike the simple dynamics of theoretical models for autoassociative memory recall [4], the dynamics of hippocampal networks implementing this function are dauntingly complex. Individual neurons change their integration properties on multiple time scales [24], [25], the activity of pyramidal cells is modulated by a plethora of functionally specialised inhibitory neurons [26], [27], each with its own intrinsic dynamics and connectivity properties[32], innervating distinct domains of pyramidal cells [28], [32] and inducing task-specific oscillations in several frequency bands [26], [28]. Here, we have shown that it is possible to dissect some of this complexity in light of the circuit approximating optimal auto-associative memory recall. Nevertheless, it should be noted that there is still more to be said about the contribution of different gross neuroanatomical features of the hippocampus (and CA3) to associative memory, as well as the roles that various cell types may play in it. Addressing these questions were outside the scope of the present study and thus remain the subject of future work.

### Distinctive features

The recall dynamics in our theory share some of the basic features of standard autoassociative memory networks (recurrent excitation, linear inhibition [4], [8]), but refine them in several critical ways. First, traditional approaches require considerable fine tuning of parameters for scenarios different from the standard Hopfield network (storing binary patterns with the additive ‘covariance’ learning rule). In our approach, the basic form of the network dynamics during recall is fully specified by the statistical properties of the recall cue and the storage process, with no free parameter left to be tuned. (Note, though, that tuning the amplitude of population oscillations in the more sophisticated, tempered transition dynamics, might be useful to improve the speed of convergence.) This allowed us to include the effects of a markedly non-uniform prior over the delay after which a memory needs to be recalled, motivated by human forgetting data [42], in contrast to traditional autoassociative memory models that assume, mostly implicitly, an (improper) uniform distribution over a finite range of recall delays (see also Text S2).

Second, our theory provides an explicit prescription for how the excitability of neurons should be regulated depending on the efficacies of both their incoming and outgoing synapses. Akin to standard approaches, this means that excitability should depend on all previously stored patterns. However, while previous proposals for adjusting neuronal excitability require a somewhat heuristic offline procedure where the full list of stored patterns needs to be known [38], we were able to show that the appropriate regulation of neural excitability in our system can be well approximated by commonly-assumed online forms of IP [60] resulting in competent recall performance.

Third, while standard approaches only consider deterministic dynamics, our dynamics are stochastic. This makes it straightforward to optimise network performance for a squared-error loss, and additionally allows for a simple representation of uncertainty, thereby also naturally accounting for hippocampal flickering phenomena (see also below).

Finally, in keeping with other statistical treatments of autoassociative memory, which, however, number only few [30], the recall cue modulates the network dynamics throughout retrieval (as an external field) rather than just being an initial condition. Its relative contribution to the dynamics reflects its quality (or noisiness).

### Storage versus recall performance

One powerful, yet biologically-untenable, simplification made by previous autoassociative memory models [4], [8]–[10], [14] is the use of additive learning rules. The memory capacity of such networks is linear in the number of synapses per neuron [1], [4], but at the cost of unrealistic synaptic and neural dynamics. At the other extreme, the importance of bounded synaptic plasticity has been investigated using synapses with extremely limited dynamic ranges, including synapses with only two states [7], [30]. While the capacity of such networks was shown to be disappointingly poor, recent work has shown that metaplastic synapses can store memories far more efficiently than synapses with the same range of efficacies but without metaplasticity [17]. As theoretical work investigating the role of metaplastic synapses in memory has so far concentrated on the benefits for *storing* information, it has been unclear how these benefits can be translated into *recall* performance – which is what ultimately matters for the organism (after all, there is not much point in storing memories if they cannot be recalled). Surprisingly, almost no work has considered the quality of memory recall from metaplastic synapses, with the notable exception of Ref. [22] who found only very modest improvements compared with the recall performance of simple two-state synapses. Thus, it had remained unclear if the benefits of metaplasticity in terms of information stored can be translated into recall performance.

We have shown that with appropriate recall dynamics, recall performance can in fact be substantially improved using metaplastic synapses (without explicit optimisation of the synaptic plasticity rule used for storage), avoiding the characteristic of simple two-state synapses that they exhibit catastrophically poor logarithmic scaling of memory life time with the number of synapses (Fig. 3). While our measures of performance are currently based on numerical simulations, it may be possible to apply and extend the analytical approaches originally developed for computing the recall capacity of simpler network dynamics [22] to provide a systematic analysis of the performance of our optimal network dynamics.

The performance of our recall dynamics follows the qualitative trends predicted by earlier analyses of metaplastic synapses [17]. However, there remain some quantitative discrepancies: for example, the cascade depth at which stored information is maximised is not the same as that at which recall error is minimised (Fig. S5). There may be several sources of these discrepancies. First, the degree to which our approximately optimal recall dynamics is able to make use of the information that is stored in the synapses may depend on the parameters of the system. Second, analyses of stored information typically quantify information in single synapses (using measures such as the signal-to-noise ratio, SNR), while recall error (e.g. fraction of correctly recalled bits for whole patterns) is a result of the information stored jointly in all synapses. These metrics may themselves only be related to each other in a complex and nonlinear manner. For example, when synaptic weights are correlated, these two information measures will differ in general. In this work, we have side-stepped this issue by using an approximation which treats synapses in the network as independent given a particular pattern has been stored. This is formally incorrect in statistical terms, as we expect dependencies between synapses sharing a pre- and post- synaptic partners. Indeed, weak but significant correlations are observed between such synapses in the cortex [68]. It will be an important next step to explicitly consider these statistical dependencies and their significance for memory retrieval [43]. More importantly, however, these measures implicitly quantify performance on fundamentally different tasks: while SNR is appropriate for measuring recognition performance, i.e. the error in making the relatively simple binary judgement on whether a particular (and noiseless) pattern has been stored in the past [17], our central interest has been recollection performance, i.e. the error on the much more demanding task of recalling the details of a high-dimensional pattern from noisy input [4], [22].

### Intrinsic plasticity

Our model predicts changes in neuronal excitability that can be traced back to the specifics of CA3 synaptic plasticity (i.e. the NMDA-receptor dependence of learning). In particular, we expect that the excitability of individual neurons should constantly change as a function of the state of the incoming and outgoing connections to and from the neuron. A range of experiments has long confirmed the homeostatic regulation of a neuron's responsiveness to injected current after chronic manipulations of network activity, corresponding to 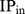 in the model [25], [33]. More remarkably, recent evidence confirmed that neuronal excitability is also modulated by the strength of a neuron's outgoing connections [57], [58], closely matching the predictions of our model for 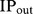: not only do the shifts in presynaptic neuron excitability follow the apparently anti-homeostatic direction predicted (increases after LTP, reduction after LTD) [57], [58], but this form of plasticity was also shown to be specific to excitatory-to-excitatory connections [58], as required by the theory. To our knowledge, we are the first to ascribe a functional role to such presynaptic IP. Furthermore, while homeostatic plasticity has been introduced in some models as a heuristic addition to the network dynamics, meant to enhance stability during learning [59], [60], here it is derived from first principles, as a necessity for optimal recall.

Our model also offers insights into some of the paradoxical findings surrounding IP. Namely, while homeostatic IP can be robustly expressed *in vitro* by pharmacological manipulations [25], as we noted, the changes in excitability reported *in vivo* after more naturalistic manipulations (e.g. after learning) are typically anti-homeostatic [23], [24] (see also [25]). Our results suggest that, although different experimental manipulations may preferentially expose one or the other mechanism (see [25], [57], [58]), both are necessary for circuit function, and that the presynaptic anti-homeostatic component dominates overall (Fig. 4D). This would explain non-homeostatic increases in neural excitability in the hippocampus after hippocampus-dependent learning [23], [24].

Our model offers an equilibrium theory – we expect constancy of neural excitability over the long-run, irrespective of the details of synaptic plasticity, at least as long as there exists a stationary distribution for the weights. Such a balance is consistent with experimental findings about spatial learning preserving the global firing rate of the network [69]. However, it is not obviously consonant with the observations of net shifts in excitability that have been measured across a population of CA3 neurons following learning [23], [24]. One possibility is that this comes from a detection bias given sparse population patterns, such as those observed in CA3 [70]. That is, for such populations, we predict that the overall balance in excitability is achieved by large increases in excitability in the small subset of neurons that are active in the pattern, accompanied by small decrements of excitability in the inactive population of neurons. If the larger changes are preferentially detected (e.g. simply due to signal-to-noise constraints in recordings), the changes in excitability that will be evident will be positive but not negative. Indeed, the pattern of experimental reports follows this trend: not all neurons recorded during the course of an experiment show detectable changes in excitability, but when they do, those changes are positive [23], [25]. The model also makes the novel prediction that differential shifts in excitability should be recorded after separating neurons based on their activity in the pattern being stored. Using indicators of immediate early expression gene (c-Fos) expression to generate a lasting tag for the neurons that are active during the encoding of a particular memory (when the animal is exposed to a novel environment [71]) should make it possible to probe the excitability of these neurons at various retrieval delays, thus directly testing our prediction for the temporal evolution of excitability following memory storage (Fig. 4D).

### Feedback inhibition

Another key prediction of our model concerns the structure of the inhibitory circuitry that provides feedback inhibition in CA3 (most likely by fast-spiking basket cells [72], [73]). In particular, optimal recall dynamics require a form of feedback inhibition that dynamically tracks excitation, but without the need for tonic levels of excitation and inhibition to be tightly balanced. This mode of operation is fundamentally different from previously proposed theories of E/I balance in cortical circuits requiring tonic excitation and inhibition to match [61], because it is the very difference between tonic excitation and inhibition levels (on the time scale of a recall trial) that carries the information about the identity of the pattern that needs to be recalled (and about the confidence in this pattern). The network is therefore operating in a rather different regime from other work on associative memory in balanced spiking networks which has considered additive synaptic plasticity [74]. In our model, when the stored patterns are sparse, this translates into inhibition dominating neural responses, as reported for sensory responses in awake (but not anesthesized) mice, at least in V1 [75]. It also predicts a net shift between excitation and inhibition within the same neuron depending on the memory being retrieved, consistent with a shift in the average membrane potential of hippocampal place cells depending on whether the animal is inside or outside their place field [76]. At a finer temporal resolution, we also expect that fluctuations in excitation and inhibition are closely correlated. There is evidence for this in neocortical recordings [75] but a test of this prediction in the hippocampus has yet to be performed.

At the level of the underlying hippocampal circuitry, the model predicts a high degree of overlap between a neuron's monosynaptic excitatory and disynaptic inhibitory partners, which could, in principle, be detected anatomically [77] or functionally [78]. Indeed, recordings in behaving rats confirm a close functional coupling between excitatory and inhibitory cell populations [79]. Moreover, as the underlying recurrent connectivity is modified, e.g. during learning, the inhibitory circuitry should be plastic as well, on a time course similar to that of learning at excitatory synapses. One recent experiment demonstrates that, at least in CA1, such structural plasticity of inhibitory connections does accompany the induction of (synaptic and structural) plasticity at the excitatory synapses [80]. At the level of synaptic plasticity, theoretical models of excitatory-inhibitory networks have already predicted the dynamical matching of excitatory and inhibitory inputs in individual excitatory cells [63]. A recent experiment found evidence for this by measuring the profile of inhibition during learning of a new spatial representation [51]. This experiment revealed a reconfiguration of inhibitory activity that mirrored the reorganization of excitatory activity during place map formation, as we would expect from a process actively matching excitation with inhibition.

### Oscillations

We have shown that a periodic modulation of the relative contribution of external versus recurrent inputs facilitates the exploration of the state space of the network, and hence improves performance when there is limited time to answer a recall query. Such periodic modulation of extrinsic vs. recurrent inputs has been anticipated to be useful in the rather specific context of sequence disambiguation [81] but its general utility for memory recall under time-pressure is a novel aspect of our model.

The computational role we ascribe to oscillations leads to a number of predictions that are unique to our theory. First, the main effect of oscillations in CA3 should be on the variability rather than the rates of pyramidal cell responses (Fig. 7C). This points to gamma oscillations as potential biological substrates because they have only weak effects on the firing rates of CA3 pyramidal cells [82]. Second, the transmission of information to read-out areas of CA3, most prominently to pyramidal cells in hippocampal area CA1, should also be strongly modulated by the oscillation, because only samples from the target distribution at the peak of the underlying oscillation (corresponding to temperature = 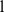) are correctly representing the pattern that needs to be recalled. This means that CA3 input into CA1 should be periodically gated such that it impacts CA1 preferentially at this phase of the oscillation, which is consistent with gamma modulation of population rates being stronger in CA1 than in CA3 pyramidal cells [82]. Further evidence for this oscillatory coordination between CA3 and CA1 is that their in-phase synchronization in the lower gamma band is a signature of coordinated memory reactivation across the hippocampal network [83], [84], and in particular of the transfer of information between them [84], [85]. This analysis does not delimit a role for theta oscillations.

Another novel prediction of the theory is that response variability across the CA3 pyramidal cell population (measured, for instance, by the entropy of their responses across trials) should depend on the phase of gamma oscillations. This can be directly tested using multielectrode hippocampal recordings in awake behaving animals, pooling data across trials in which the same item is being recalled (e.g. the same spatial position is being traversed), and measuring the variability across such trials as a function of the phase of the simultaneously recorded gamma oscillation.

Mechanistically, our model of oscillations requires a rhythmic modulation of the different excitatory inputs to pyramidal cells in CA3, affecting the relative contribution of recurrent versus perforant path inputs. While the specific mechanisms achieving this effect remain unclear, recent evidence suggests that at least two classes of inhibitory neurons – bistratified [26] and oriens-lacunosum moleculare (OLM) cells [86] – can rhythmically modulate external versus recurrent inputs to pyramidal cells, as would be required in our model. As OLM cells show strong modulation by gamma [87], they seem to be ideally placed to play this role.

Lastly, our analysis of retrieval performance revealed an inherent tradeoff between the utility of oscillations when exploring complex posterior distributions (very wide for old patterns or multimodal as in the case of the flickering experiment) and their detrimental effects when the correct answer is very clear (the posterior is sharp and unimodal, as for recent patterns). It is tempting to speculate that the amplitude of gamma oscillations could be modulated with task difficulty (estimated by some measure of response confidence, which is readily provided in a sampling based representation) to optimise retrieval performance. Indirect evidence for this comes from chronic recordings in the human hippocampus showing increased gamma (and theta) power for retrieving remote versus recent autobiographical memories [88].

### Representation of uncertainty

One key aspect of our theory is that the uncertainty about the patterns that are being recalled is represented along with the patterns themselves. This facilitates recall within the network, and it is also essential for downstream functions such as decision-making, for which evidence from recalled memories has to be combined with other, e.g. perceptual, sources of information – weighting each source of information with their respective certainties [45]. The behavioural ability to assess confidence in a retrieved memory trace has been demonstrated in various species, including humans [89], [90]. We proposed that this is underpinned by a sampling-based neural code for uncertainty in the hippocampus [45], [91]. Although the neural dynamics considered here are highly simplified, recent theoretical work has shown that the dynamics of more realistic leaky integrate-and-fire neurons can closely approximate those required by Gibbs sampling used here [54].

We showed that a sampling-based representation can explain some puzzling experimental observations revealing transient flickering in population responses following an instantaneous transformation of the spatial context [46]. In order to capture this flickering in traditional attractor dynamics, high levels of input noise would need to be assumed. However, in the actual experiments, special care was taken to make the cues for the individual environments as reliable as possible, so that the animals faced a problem of ambiguity rather than noise. According to our theory, hippocampal flickering is a variant of bistable ‘spatial perception’, and as such can be viewed as a signature of the dynamics exploring different modes of the posterior, each corresponding to one of the stored memories. Bistability poses a particular challenge to attractor dynamics, which actively eliminate ambiguity by a winner-take-all mechanism. Conversely, sampling-based representations have been used to account for a host of perceptual and neural phenomena surrounding bistable perception [45], [92]–[95]. If our sampling-based interpretation of flickering is correct, then it should be possible to modulate the degree of flickering, and the distribution of dwell-times for the individual representations, by experimentally manipulating sources of uncertainty (the reliability of sensory cues, or the prior probabilities of the animal finding itself in any one of the possible environments).

### Conclusions

In sum, our work makes two important contributions. First, it shows for the first time that high-quality recall from metaplastic synapses is at all possible with neurally plausible dynamics. Second, the resulting recall dynamics involve several critical motifs that had not been predicted by standard approaches, and yet map onto known features of hippocampal dynamics. Thus the model provides insights into the computational role of several aspects of hippocampal activity and allows us to make a range of novel, experimentally testable, predictions.

## Methods

### Pattern and input statistics

We model a network of 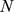 neurons, with connectivity defined by matrix 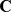, with 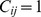 if there is a synapse from neuron 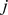 to neuron 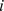 and 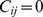, otherwise. To control connectivity (Figs. 2E, 5E–F, and 6), a randomly selected 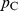 fraction of elements in 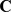 was set to 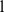 and the rest to 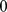. The corresponding synaptic efficacies are binary and defined by matrix 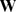, which is obtained as the result of storing a sequence of patterns 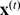 by the cascade learning rule (see below). The patterns are also binary and, consistent with data suggesting that the inputs to the CA3 network are decorrelated by the dentate gyrus [96], we assume individual bits in a pattern to be independent, such that the distribution of the stored patterns factorizes over neurons (and also, implicitly, over patterns):
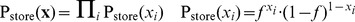
(9)where 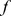 is the pattern density, or coding level.

Finally, the recall cue is a noisy version of the original pattern, corrupted by independent noise modelled as a binary symmetric channel:
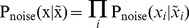
(10)

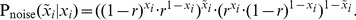
(11)with parameter 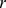 describing the probability of a bit in the original pattern being flipped in the recall cue.

Pattern age 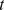 is assumed to be distributed geometrically with mean 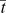:
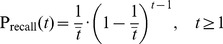
(12)


### Cascade rule

Learning is stochastic and local, with changes in the state of a synapse 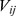 being determined only by the activation of the pre- and postsynaptic neurons, 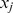 and 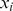 and the current value of 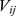. Following the presentation of a pattern with activation 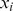 and 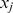, the synaptic state transitions from the current state 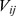 to to the new state 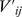. In the most general form, the probability of a synapse changing between any two states can be defined through a set of transition matrices 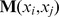, with 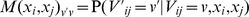, which leads to a large number of model parameters. A natural way to reduce this number is to define a transition matrix for potentiating, 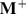, and depressing, 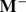, events and separately map different neuron activation pairs into such events, possibly with some pairs leading to no change. Here, we assume a postsynaptically-gated rule, where the co-activation of pre- and post- neuron leads to potentiation, while an active postsynaptic neuron causes depression if the presynaptic neuron is silent, i.e. 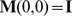, 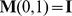, 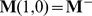, 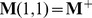, with 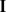 denoting the identity matrix. For comparison, we also use the traditionally assumed presynaptically-gated learning rule [22], [38], with 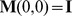, 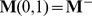, 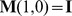, 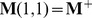.

We express the two transition matrices 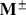 using a generalization of Fusi et al.'s 2005 cascade model [17], parametrized by 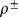, 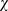 and the cascade depth 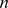. We index states corresponding to weak and strong synapses with 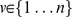 and 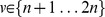, respectively (Fig. 1C). We describe the elements of the transition matrix, 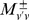, as a sum of two terms: 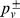 describing the probability that a weak (strong) synapse in state 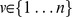 (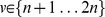) will potentiate (depress) to become a strong (weak) synapse, by occupying the ‘shallowest’ corresponding state in the cascade hierarchy, 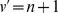 (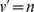); and 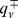 describing the probability that a weak (strong) synapse in state 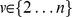 (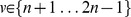), will remain weak (strong), but even more so, by changing to a corresponding state that is one step deeper in the cascade hierarchy, 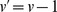 (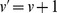).

The probability of potentiation and depression decays as a geometric progression: 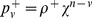 for 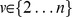 (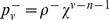 for 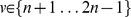), and we set 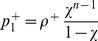 (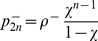) to compensate for boundary effects. The probability of transitions towards deeper metastates is defined as 
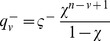
 for 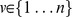 (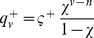 for 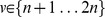) with the correction parameter 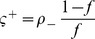 (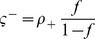) ensuring that different metastates are equally occupied for any pattern sparseness value 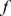, as done in the original model [17]. Additionally, the constraint 
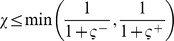
 ensures that we have proper transition probabilities for 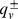. The two additional parameters 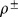 are inspired by previous work on simple binary synapses, which showed that, for sparse patterns, it is beneficial to have different transitions probabilities for potentiation and depression [10]. The original Fusi model [17] can be easily recovered by setting 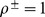, 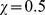.

#### Computing the synaptic weight distribution

A key quantity that is determined by the synaptic plasticity rule, and that we will need for deriving the optimal recall dynamics below, is the probability, 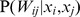, that the weight of the synapse between presynaptic neuron 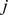 and postsynaptic neuron 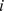 takes a particular value after having stored pattern 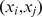 some time ago, where the delay (time since storage) is drawn from the prior over pattern ages, 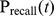. For this, we first need to understand how the probability distribution of the underlying synaptic state, 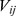, evolves over time.

The presentation of a sequence of patterns (intervening between storage and recall time) drawn independently from 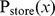 defines a Markov process, described by a transition matrix 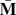. This matrix defines the transition probabilities caused by the storage of an individual intervening pattern, obtained by marginalizing over the unknown identity of this intervening pattern:
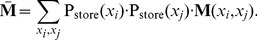
(13)This transition matrix also defines the stationary distribution of the synaptic states 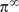 as the eigenvector of 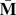 corresponding to the eigenvalue 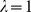, with 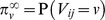 under the stationary distribution (with 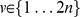).

Using this notation, the evolution of a synaptic state after encoding a pattern 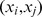, reduces to a sequence of matrix multiplications, starting from the stationary distribution, 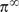 (corresponding to having stored an infinite sequence of patterns prior to storing 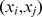), applying the transition induced by the pattern 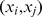, then applying repeatedly the operator 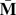 the appropriate number of times. Formally, the distribution over the synaptic states, for pattern age 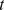 (i.e. after storing 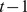 intervening patterns), can be expressed as:
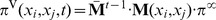
(14)where 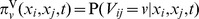. An example of the evolution of this distribution under cascade dynamics, when the stored pattern is 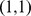 is shown in Fig. 1D.

Next, as the pattern age 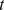 is unknown at the time of recall, we need to integrate over all possible pattern ages, with probabilities given by 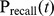:
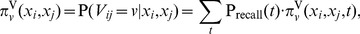
(15)


To make the marginalisation of the unknown pattern age 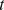 practical, we use the diagonalized form of the transition matrix 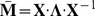, with 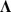 being a diagonal matrix containing the eigenvalues of 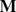, and 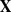 a matrix having the corresponding eigenvectors as columns. As the expression in Eq. 15 is linear, we can reorder the operations and compute Eqs. 14–15 in a single step:
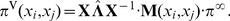
(16)where 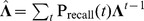. It is hard to compute the eigenvalues and corresponding eigenvectors analytically in general; thus, we estimate them numerically. Nonetheless, if the prior over 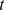 is relatively simple, it is possible to do the marginalization analytically, with 
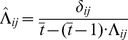
 for the geometric prior we used in our simulations (see above), where 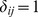 for 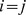, and 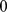 otherwise.

Finally, we use the deterministic map of synaptic states into synaptic efficacies (formalised as a 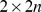 matrix 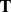, with 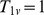 for 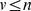; 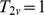 for 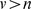; and 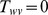, otherwise) as:
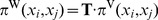
(17)where 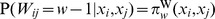 with 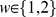.

Note that this approach is general and can be applied to any synaptic plasticity model which involves stochastic transitions between a finite set of states, e.g. the serial model of Ref. [55].

### Optimal recall

As is conventional, and plausibly underpinned by neuromodulatory interactions [97], we assume that network dynamics do not play a role during storage, with stimuli being imposed as static patterns of activity on the neurons; and conversely, that the network does not undergo further plasticity during recall.

#### The posterior distribution over patterns

A recall query implies a posterior distribution over patterns, given the information in the weights and the recall cue:
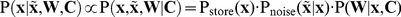
(18)The first two terms composing the posterior have been defined in the section describing ‘pattern and input statistics’ above. To be able to analyze the last term, we make the approximation of assuming that the evidence from the synaptic efficacies factorizes over individual synapses as 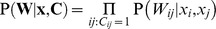, where we have derived the form of the individual terms, 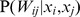, in the preceding section. To simplify notation and since we usually focus on all-to-all connected networks, the dependence on matrix 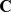 is not made explicit in the main text.

Note that we do not assume that this posterior is ever computed explicitly by a neural circuit: we use it merely as an intermediate conceptual step to construct network dynamics that produce activity patterns optimizing network performance under this posterior distribution.

#### Gibbs sampling

All procedures that we use for sampling from the posterior distribution in this paper are variations of Gibbs sampling which updates sequentially dimension (neuron) 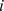 of the vector 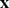, conditioned on the current state of all other dimensions (neurons), 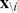, by sampling from 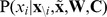. For binary variables, as in our case, this is equivalent to computing the log-odds ratio:
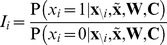
(19)and then passing it through a logistic sigmoid nonlinearity 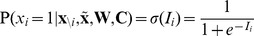.

Under the assumptions used for computing the posterior described above, the log-odds ratio can be decomposed into individual contributions from the prior over patterns, from the recall cue, and from individual synapses in the network that link neuron 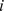 to its pre- or postsynaptic partners (by applying Bayes rule, and appropriately ordering the factors):
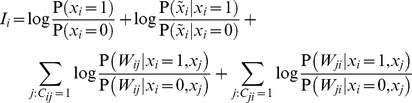
(20)


The contributions of individual recurrent weights in the total current is computed using the expression for 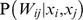 in Eq.17. This results in a set of 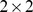 possible outcomes depending on the value of the synaptic efficacy 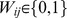 and presynaptic activity 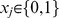: 
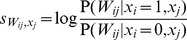
, which can be further rewritten as a quadratic form in the two variables, 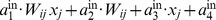, with the parameters 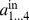 computed as 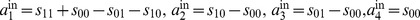. A very similar functional form can be obtained for the outgoing synapses. Note that these values are thus fully determined by the parameters of the learning rule (here, the cascade rule), the pattern distribution, and the prior for the pattern age, with no free parameters. (These parameters can also be derived for the case when the true pattern age is known, by replacing the prior for the pattern age with a delta function in Equation 16.) As a special case, when using a balanced (for which the average probability of a potentiation or depression event is the same) presynaptically-gated learning rule we find that 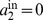; equally, by symmetry, for a balanced postsynaptically-gated learning rule we will have 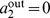.

Putting everything together, the total current to a neuron under Gibbs dynamics has the form:
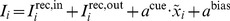
(21)

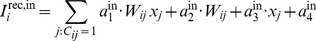
(22)

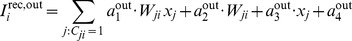
(23)where 
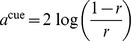
 and 
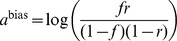
.

Starting from the recall cue, the recall dynamics involve asynchronous updates of each neuron in the network, with samples collected at the end of each full network update, corresponding to one time step in the figures. The permutation determining the order in which neurons are updated is also randomly redrawn at the beginning of each network update.

### Network approximations

#### Intrinsic plasticity

We consider three variants for approximating the term corresponding to the outgoing synapses (presynaptic IP):
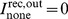
(24)

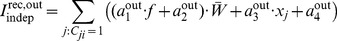
(25)

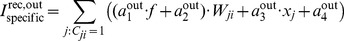
(26)Computationally, 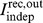 corresponds to an approximation of the expected value of 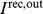, conditioned on the net activity of the neuron's postsynaptic partners, 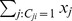. The last, most refined approximation, 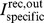, represents a similar expectation, further conditioned on the sum of the efficacies of outgoing synapses, 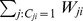. We obtain 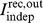 by taking an expectation over 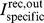 (implicitly still ignoring correlations between weights and postsynaptic activities).

To investigate the role of the homeostatic regulation of neural excitability depending on the incoming synaptic weights (postsynaptic IP), we replaced the term corresponding to 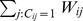 by its expected value, 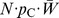, with 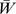 the expected synaptic efficacy under the stationary distribution, and 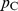 the synaptic connection probability (see above). Furthermore, when introducing or removing the homeostatic regulation of excitability in Fig. 4A (bottom) we replace 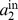 by 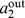 (alternatively, we could have varied the factor scaling the dependence of the total synaptic efficacy parametrically).

For both forms of regulation of neural excitability, the online version of the recall dynamics assumes the term 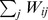, or 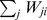, respectively, is replaced by a temporal average of the form 
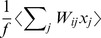
, with the presynaptic activity sampled from the prior, which would correspond to network activity while retrieving other patterns (we use a square temporal window and only vary its width, i.e. the number of samples used for the estimation, but a more realistic time decaying kernel would also be possible).

#### Inhibition

To model tonic inhibition, we replace the inhibition terms 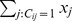 and 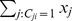 by their expected value 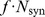, with 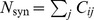 (or 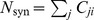) the total number of pre- or postsynaptic connections to neuron 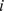. To asses the importance of spatial selectivity of inhibitory connections we use a sparsely connected network (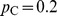) and vary the degree of overlap of the sources of excitation and inhibition to a neuron, while maintaining the average magnitude of inhibition fixed. In particular, we define a second connectivity matrix 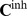 for defining the sources of (disynaptic) inhibition to each neuron and vary the E/I overlap by manipulating the similarity between 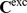 and 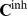. To preserve the average net inhibition to a neuron, we keep the average number of inhibitory connections to a neuron fixed (the two connectivity matrices are equally sparse), and replace a certain percentage of the correctly matched inhibitory sources to a neuron (e.g. in Fig. 5D from neuron 4 to neuron 2) with inhibitory connections from neurons that are not in the set of its (pre- or post-) synaptic partners (e.g. from neuron 5), with the E/I overlap parameter defining the probability of a ‘correct’ inhibitory source actually feeding inhibition to a neuron, 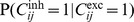. Furthermore, to keep the net inhibitory current to the neurons unchanged, we add random inhibitory connections from neurons that do not share recurrent collaterals with neuron 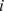 as to preserve the average number of inhibitory synapses onto the neuron. Using this setup, the feedback inhibition term becomes 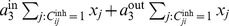.

### Artificial dual sampler

We construct artificial recall dynamics that perform Gibbs sampling in the space of the joint distribution 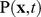 [49]. Introducing the pattern age as an auxiliary variable in the sampling procedure can be related to other auxiliary variable methods for sampling and is expected to improve sampling efficacy in the case of complex distributions [65].

Formally, the dynamics alternates between sampling an individual neuron's activity, conditioned on everything else (including the current value to 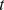), 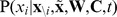 and sampling the pattern age 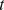, according to the distribution 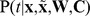 (which simplifies to 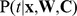, as 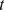 is independent of the recall cue after conditioning on the stored pattern 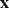). This last step makes this sampling procedure biologically unrealistic, as computing the distribution over pattern ages requires knowledge of the full set of recurrent collaterals of the network.

Practically, the procedure involves stochastic updates of neuron activities that are very similar to those of the simple Gibbs sampler, with the distinction that now the parameters 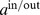 depend on the pattern age, 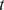, and are computed using the distribution 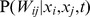, obtained by projecting the distribution over the synaptic states 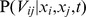 directly into synaptic efficacies, without marginalising out 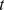. This means that the expression of the total current Eq. 3 now includes age-dependent parameters 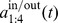. Conceptually, this will result in a modulation of the relative contribution of the recurrent collaterals versus the external input from the cue, such that the recurrent dynamics dominate for recent patterns while the output is driven by the external input when the pattern is deemed to be old, when little or no information about the pattern is available in the weights.

Finally, to be able to sample the pattern age, we limit the maximum possible pattern ages resulting in a finite discrete distribution, from which it is easy to sample (in practice, we assume events with 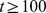 can be treated as equivalent). As there is little signal in the tail of the distribution over pattern ages, this does not affect performance for the network sizes considered here.

### Tempered transitions and network oscillations

Tempered transitions (TT) is a method that can improve sampling efficiency by using annealing, i.e., systematically increasing and then decreasing a temperature parameter to ensure better exploration of the state space [64]. According to TT, in order to sample from a target distribution 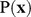 we choose a set of intermediate probability distributions, 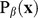, indexed by the inverse temperature parameter 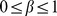, that are increasingly dissimilar from, but also easier to sample than, 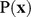 as 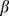 decreases. The target distribution is represented at inverse temperature 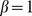: 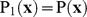. For each intermediate distribution we need a form of stochastic dynamics (formally, defining a Markov transition operator) which samples from (i.e. has as its stationary distribution) the corresponding 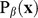. Starting from the current state 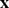, which is a sample at 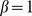, i.e. from 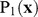, a sampling cycle involves first lowering the inverse temperature in a sequence of steps down to 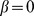 and then increasing it back to 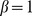. At each temperature level, we run the corresponding stochastic dynamics for a few steps starting from the last sample collected at the previous temperature level. This results in a sequence of intermediate samples, 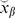 and 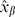, for the descending and ascending inverse temperature sequences, respectively (Fig. S4). Finally, all the intermediate samples produced at inverse temperatures 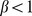 are discarded, and the final sample produced at 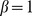 is accepted or rejected (in which case network activity would need to return to the state it had at the beginning of the cycle) with a probability given by the product of pairwise ratios of probabilities of all the intermediate states [64] (see also Suppl. Info. in Ref. [49]).

For us, the target distribution is the posterior 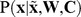. Common practice would dictate that we choose the intermediate distributions to be simply exponentiated (with 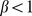 as the exponent) versions of the target distribution, which would result in a completely uniform distribution at 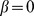. However, this would not be efficient as the uniform distribution has no information about the original problem and thus results in unnecessarily wide Markov steps and, as a consequence, in a high rejection rate. Instead, we can use an important insight about the structure of our posterior to construct a better sequence of intermediate distributions. This insight is that the only factor that makes the posterior hard to sample from (thus motivating the usage of TT in the first place) is the correlations in it that are solely introduced by the weight-likelihood term, 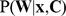. (Note that although we approximated this term above as factorized over the elements of 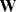, this still does not mean that it also factorizes over the elements of 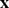, of which the correlations are of issue here.) Therefore, we chose only this term to be modulated by temperature, such that
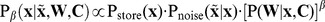
(27)


The are two important features of exact TT dynamics that are problematic in the context of our network dynamics: first, the order in which neurons are updated in the ascending phase should be the exact reverse of that used in the descending phase; second, and more critically, an acceptance step is required at the end of each temperature cycle, as we saw above. As both the final acceptance step and the tight control on the ordering of neural updates are biologically unrealistic, the neural network approximates TT dynamics by ignoring sample rejections and by updating neuron activities in a random order during an oscillation cycle [64]. Under these approximations, the network dynamics are essentially identical to those of simple Gibbs (Eqs. 21–23), with all parameters unchanged, with the only modification that the recurrent currents are multiplicatively modulated by the inverse temperature 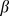 (cf. Eq. 21):
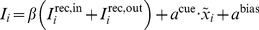
(28)At 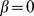 this is equivalent to sampling from a purely feed-forward network which uses no information in the recurrent weights and which is the network that we used throughout the paper as our ‘control’.

In general, the inverse temperature parameter 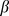 can take values between 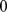 (corresponding to control) and 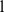 (the target distribution). Here, we took a sequence that linearly interpolated between 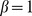 and a minimum value 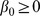, with the amplitude of the oscillation being defined as 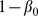. In all cases the number of neurons updated at each temperature level was chosen such that the total number of neurons updated over a whole cycle was the number of neurons in the network, 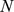.

Although, due to the approximations we introduced above, the resulting network dynamics is no longer guaranteed to generate samples from exactly the correct posterior distribution, simulation results suggest that this approximation does not significantly alter the estimate of the posterior mean or the average response variability provided that the acceptance probability under the exact dynamics remains high, which we ensure by an appropriate modulation of 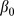.

### Simulation parameters

We start by defining the general setup and the default parameters used in all simulations, after which we proceed to list the parameter settings specific to each figure, in the order in which they are included in the main text. Unless otherwise specified, we considered a network of 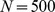 fully-connected neurons. The stored patterns were balanced, 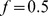 (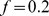 when sparse patterns were used, in Fig. 2); the recall cue noise was 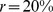, the average pattern age was 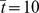 and the cascade parameters were 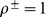, 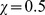 and depth 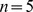.

For measuring retrieval performance, we started from sampling the stationary distribution of the synaptic states, then we sampled from the prior, 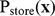, one N-dimensional binary pattern 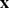 which we stored by modifying synaptic states in the network according to the cascade learning rule described above. To separate the effects of synaptic correlations from the correlations among recalled activities, we simulated the effects of the storage of intervening patterns following the storage of 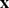 by evolving individual synapses independently for 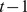 steps according to the transition operator, 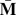, corresponding to storing a random pattern from the prior. At recall, we sampled a recall cue, which was a noisy version of 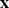, according to the noise model. This cue was provided as input to the network throughout retrieval as well as the starting point for the network dynamics. The network was allowed to evolve for 100 steps according to the dynamics we derived above. We took the temporal average across all these samples to be the recalled pattern 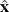, and computed the root mean square error between the stored and recalled pattern as 
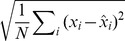
. The performance for the control feed-forward network could be computed analytically (as both the prior over 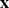 and the recall cue distribution are factorized) as 
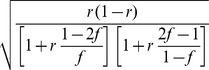
, which for the case of balanced patterns (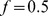, as in most cases considered here) reduces to 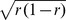. When plotting mean performance as a function of pattern age, we used 10 trials for estimating the error for each 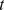; for the average performance plots, we repeated the storage-retrieval procedure described above 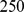 times, with pattern ages drawn randomly from the prior distribution. Average performance was measured as the average error over these independent runs, with all error bars representing the standard error of the mean.

For Fig. 2B, we stored the 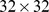 binary pattern shown and used Gibbs dynamics without further approximations to retrieve it at a pattern age 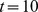. To emphasize the stochastic aspects of the dynamics, we chose to show a subset of neurons whose activity evolution happened to be most variable. When investigating the dependence of the networks' performance on the total number of synapses per neuron in the sparse condition, we fixed network size and varied the connection probability, 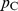; in particular, we used a network size of 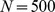 with one exception: we took 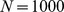 for the case of 500 synapses/neuron. To investigate the effects of the prior over pattern ages, we reran the same procedure for different settings of the average pattern age (adjusting the parameters of the network accordingly and resampling the pattern ages for which retrieval is performed).

For the memory capacity analysis in Fig. 3, we used the default parameters for the cascade (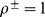 and depth 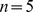) and optimized the parameters of the two-state synapse such that the signal decays exponentially with the same time constant as the prior assumed over patterns (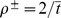 and 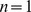). The memory capacity was defined, in line with classic SNR-based analyses, as the maximum pattern age for which retrieval error (averaged over 100 trials for each 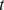) was below a predefined threshold 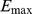. The network evolved by simple Gibbs dynamics (assuming 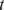 unknown). When optimizing cascade depth (Fig. S5), we assumed 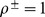, and estimated for each setting of 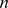 the average retrieval error under the prior for 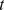 and the mutual information between a synapse 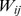 and the activity of its corresponding neurons 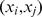 (marginalizing over the unknown 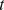; i.e. using Eq. 17).

For Fig. 5A, we monitored the excitatory, 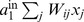, and inhibitory, 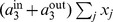, input to a neuron in a network with the default parameters settings, evolving by Gibbs dynamics, as described above (the actual values obtained were discrete so we added a small amount of Gaussian jitter to them for visualization purposes). These two quantities are plotted against each other for three example neurons, two with low entropy (red, blue) and one with high response entropy (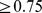). All-to-all connectivity was used for panel B, and sparse connectivity (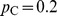) for panels E and F. Panel E shows the total recurrent current to an example neuron using the exact vs. the approximate expression for computing the inhibitory current, while the dynamics evolve by Gibbs.

For Fig. 6, we used relatively dense connectivity (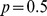) in order to preserve a relatively high number of synapses per neuron. The parameters for different approximations were 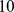 steps for the online forms of IP, and we used 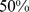 E/I coherence and oscillation amplitude 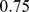.

For the simulations with oscillations (Fig. 7), we used 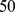 different temperature levels, linearly spanning the range 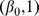, with 5 neurons updated at each temperature step, such that one oscillation cycle corresponded to one full network update (50 descending and 50 ascending inverse temperature steps), as before. In this case, the posterior mean was computed by averaging over the samples obtained at (inverse) temperature 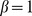. (We kept the total simulation length constant, which meant that we had a reduced number of samples for estimating the posterior with oscillations, thus slightly favoring simple Gibbs dynamics without oscillations, but we deemed this a fair comparison if the duration of a recall trial is the real constraint). For Fig. 7C, we used high amplitude oscillations, 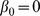, and, for each temperature level (which defines the phase of the oscillation), computed average population firing as 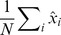 and the average response entropy as 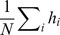, with a neuron's response entropy defined as 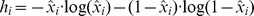.

For the flickering experiment (Fig. 8), we stored two consecutive patterns, 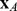 and 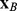 (corresponding to the two contexts in the original experiments), and simulated the effects of having stored another 8 successive patterns independently across synapses as described above. For creating inputs to the network, cues were sampled independently in each time step from the input distribution conditioned on the pattern (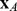 or 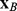) corresponding to the current context, and hence their statistics changed abruptly at a context switch. For recall, we used oscillatory dynamics (as in Fig. 7, with 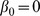) with one minor modification: instead of taking a single relatively reliable recall cue as the input, each neuron integrated the evidence from the most recent past of several highly unreliable cues (75 cues, each with 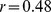) by simply summing them up (this is optimal in our framework under the assumption that all 75 cues are i.i.d., which is violated at a context switch). For constructing the actual figure, we started the simulations using 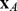 and switched to 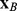 at time 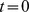, marked by the vertical green bar. As the effective recall cue was obtained by integrating over a period of several time steps, there is a corresponding time-window after the switch during which this effective recall cue is ambiguous (due to the integration of conflicting evidence coming from two different contexts), and hence the posterior is determined primarily by the evidence from the weights, which is inherently multimodal. We computed the correlation between the response of the network (at the peak of the oscillation, corresponding to 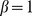) and the two actually stored patterns 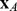, and 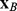, which are displayed in Fig. 8.

## Supporting Information

Figure S1
**Different schemes for representing the posterior through recall dynamics.** **A.** Schematic representation of possible strategies for constructing recall dynamics corresponding to the posterior (heat map): starting from the recall cue (green), maximum a posteriori (MAP, black line) dynamics follow the local gradient to a possibly local maximum of the posterior thus exhibiting attractor dynamics; sampling based dynamics (MCMC, gray dots) move stochastically in the state space, such that the amount of time spent in a certain region of the state space is proportional to the mass of the distribution in that region. For the purposes of illustration, the case of analog patterns is shown. **B.** The corresponding neuronal transfer functions (the expression for the total current to a neuron is identical in all variants, see Eq. 3). **C.** Comparison of retrieval performance using different retrieval dynamics. Control level was 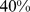 (not shown). All simulation parameters had the default values, as defined in the main text.(TIF)

Figure S2 **Representing recall uncertainty.** Relationship between the variability of neural responses during retrieval, measured by the average neural response entropy as shown in Fig. 7C, and the final (r.m.s.) retrieval error associated with the response. Colors label the age of the pattern to be retrieved (see color bar on right). Simulation used default parameters (see Methods).
(TIF)

Figure S3 **Recall performance for standard attractor dynamics.** A total of 10 patterns was stored in a recurrent network by the cascade rule, either the pre- (blue) or the postsynaptically gated form (red). All parameters were set to their default values. Retrieval followed standard attractor dynamics which ignore the prior over pattern ages and the recall cue – beyond the initial condition (see Text S2 for details). Gray dashed line shows retrieval performance for the optimal dynamics (without approximations). (This performance is formally identical for pre- and post-synaptically gated plasticity.) Black dashed line shows the usual control level, corresponding to an optimized feedforward network.
(TIF)

Figure S4
**Oscillations as tempered transitions.** Schematic depiction of the effects on the posterior induced by modulating the temperature parameter for a one-dimensional analog distribution. Tempered transitions cycles through several distributions indexed by the inverse temperature parameter 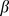 taking values between 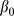 (depending on oscillation depth) and 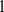. Sampling at the high temperature (low 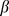) distributions allows the dynamics to explore the full state space.(TIF)

Figure S5 **Single synapse signal vs. recall performance.** Mutual information between pre- and postsynaptic activity at a synapse and the weight of that synapse (gray) and recall performance in the network (black) as a function of cascade depth. Arrows show optima of the two curves.
(TIF)

Text S1 **Alternative recall dynamics.**
(PDF)

Text S2 **Comparison to standard attractor dynamics.**
(PDF)

Text S3 **Error rates for old patterns.**
(PDF)
