# Supplementary figures and images for: Optimal Recall from Bounded Metaplastic Synapses: Predicting Functional Adaptations in Hippocampal Area CA3
Source: PLoS Comput Biol. 2014 Feb 27;10(2):e1003489. doi: 10.1371/journal.pcbi.1003489 (PMC3937414; doi:10.1371/journal.pcbi.1003489)

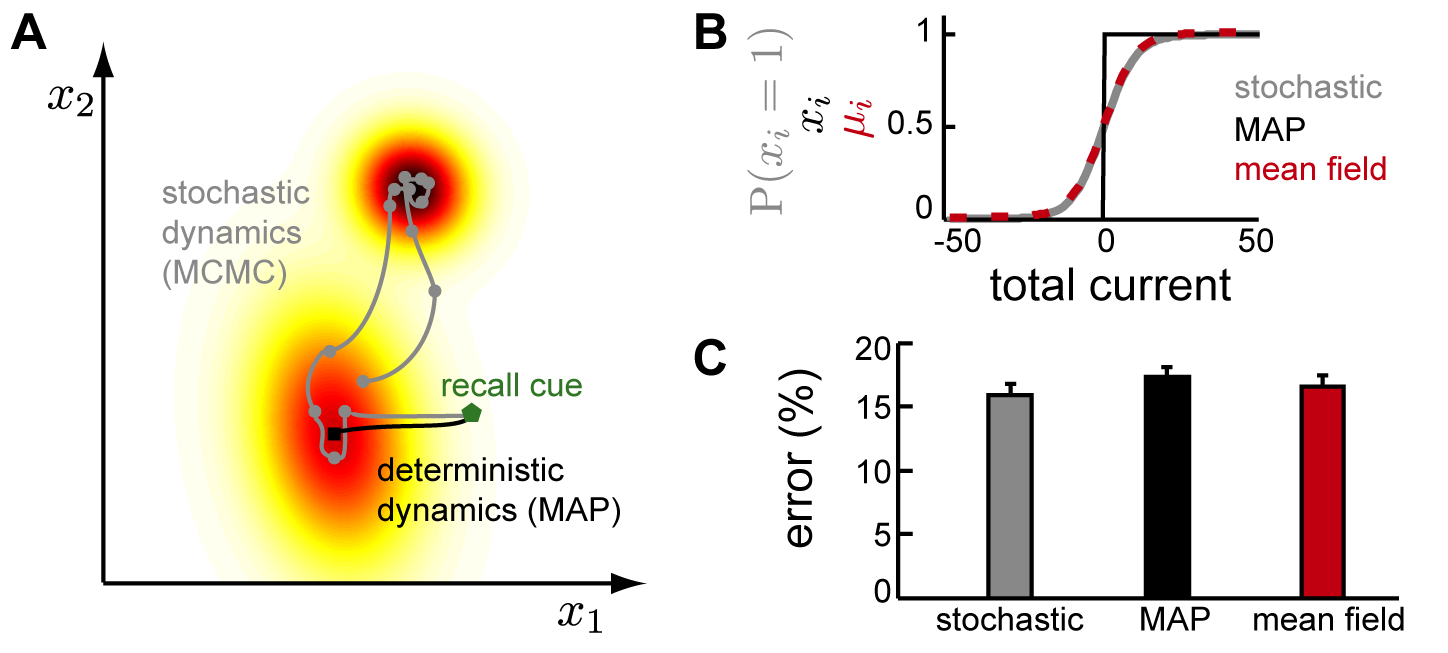

Supplement: Figure S1 — Different schemes for representing the posterior through recall dynamics. A. Schematic representation of possible strategies for constructing recall dynamics corresponding to the posterior (heat map): starting from the recall cue (green), maximum a posteriori (MAP, black line) dynamics follow the local gradient to a possibly local maximum of the posterior thus exhibiting attractor dynamics; sampling based dynamics (MCMC, gray dots) move stochastically in the state space, such that the amount of time spent in a certain region of the state space is proportional to the mass of the distribution in that region. For the purposes of illustration, the case of analog patterns is shown. B. The corresponding neuronal transfer functions (the expression for the total current to a neuron is identical in all variants, see Eq. 3). C. Comparison of retrieval performance using different retrieval dynamics. Control level was (not shown). All simulation parameters had the default values, as defined in the main text. (TIF) [file pcbi.1003489.s001.tif]

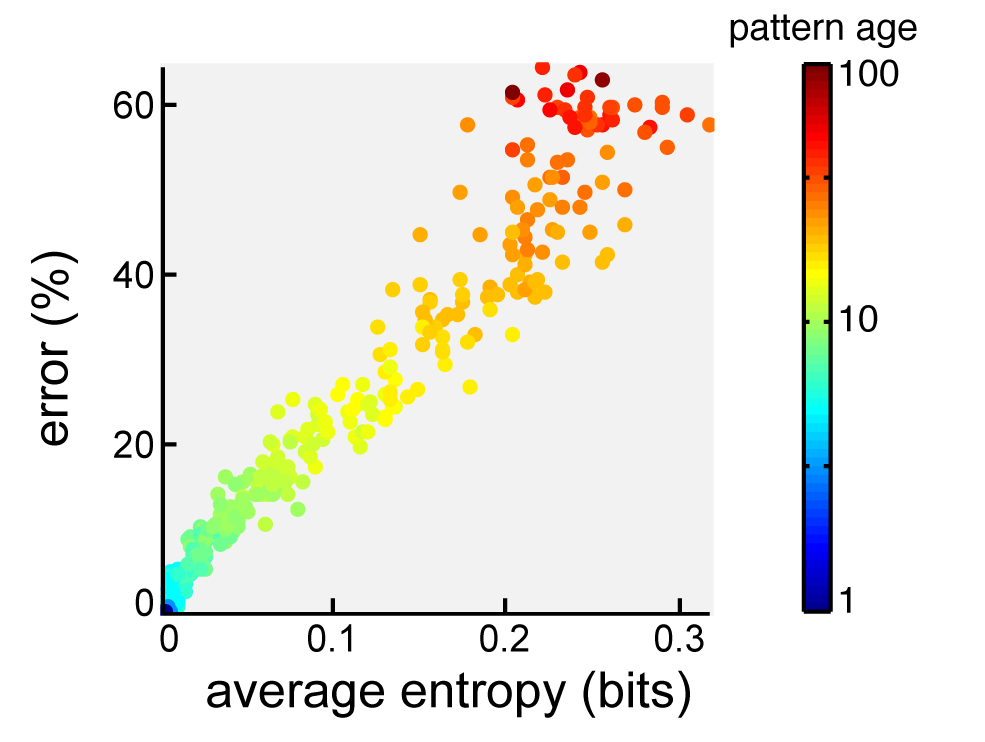

Supplement: Figure S2 — Representing recall uncertainty. Relationship between the variability of neural responses during retrieval, measured by the average neural response entropy as shown in Fig. 7C, and the final (r.m.s.) retrieval error associated with the response. Colors label the age of the pattern to be retrieved (see color bar on right). Simulation used default parameters (see Methods). (TIF) [file pcbi.1003489.s002.tif]

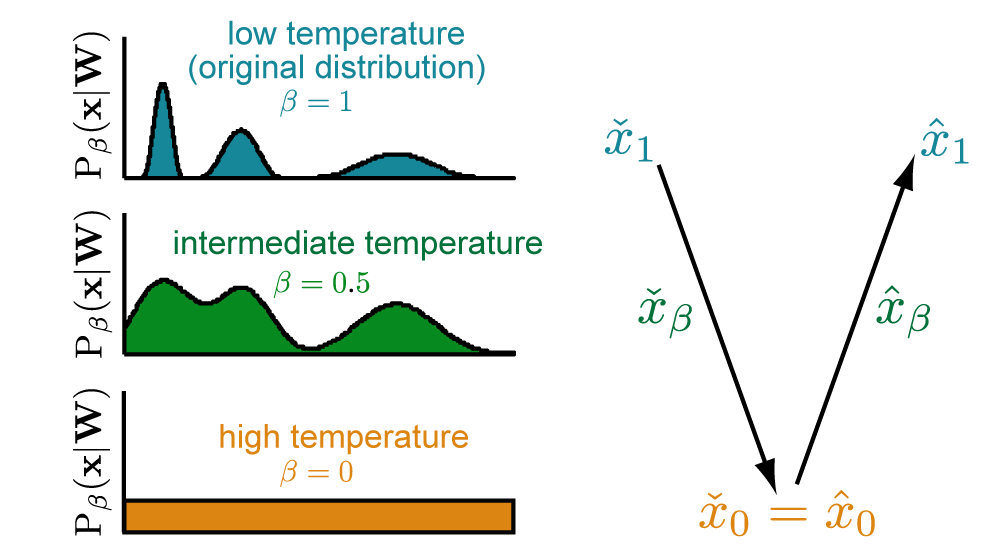

Supplement: Figure S3 — Recall performance for standard attractor dynamics. A total of 10 patterns was stored in a recurrent network by the cascade rule, either the pre- (blue) or the postsynaptically gated form (red). All parameters were set to their default values. Retrieval followed standard attractor dynamics which ignore the prior over pattern ages and the recall cue – beyond the initial condition (see Text S2 for details). Gray dashed line shows retrieval performance for the optimal dynamics (without approximations). (This performance is formally identical for pre- and post-synaptically gated plasticity.) Black dashed line shows the usual control level, corresponding to an optimized feedforward network. (TIF) [file pcbi.1003489.s003.tif]

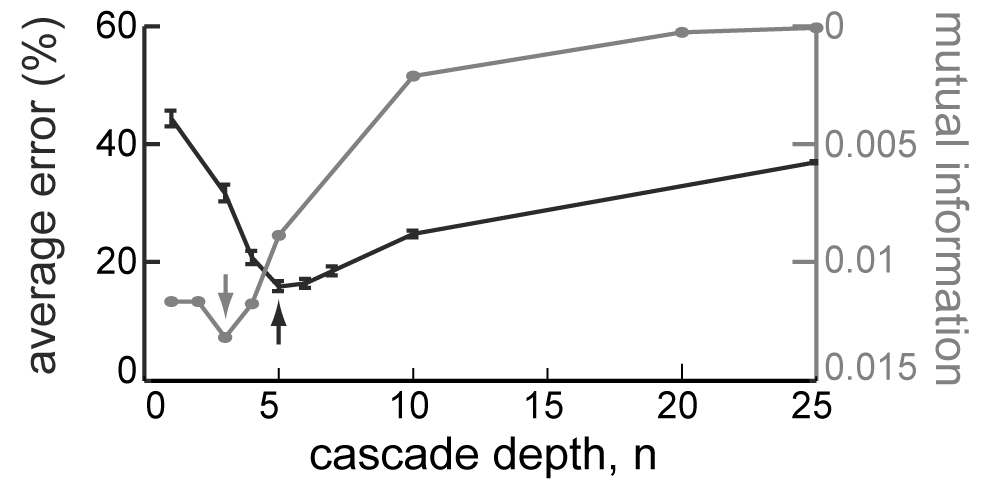

Supplement: Figure S4 — Oscillations as tempered transitions. Schematic depiction of the effects on the posterior induced by modulating the temperature parameter for a one-dimensional analog distribution. Tempered transitions cycles through several distributions indexed by the inverse temperature parameter taking values between (depending on oscillation depth) and . Sampling at the high temperature (low ) distributions allows the dynamics to explore the full state space. (TIF) [file pcbi.1003489.s004.tif]

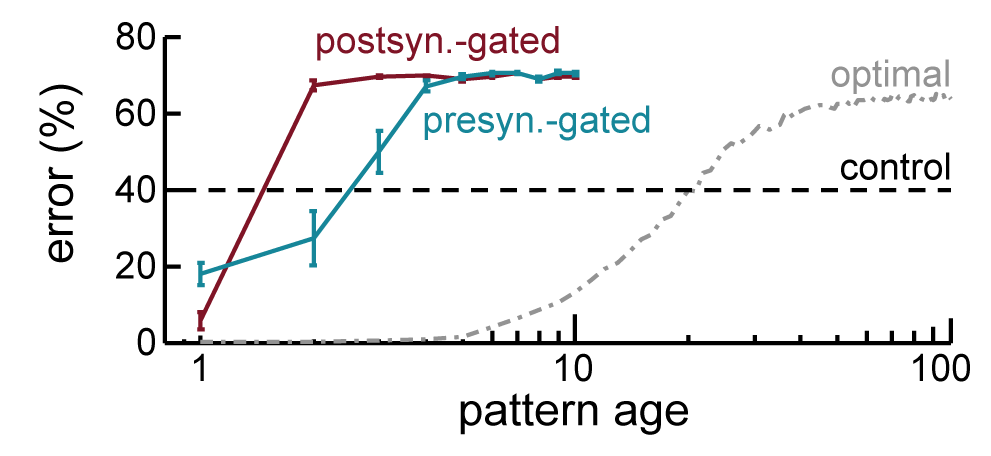

Supplement: Figure S5 — Single synapse signal vs. recall performance. Mutual information between pre- and postsynaptic activity at a synapse and the weight of that synapse (gray) and recall performance in the network (black) as a function of cascade depth. Arrows show optima of the two curves. (TIF) [file pcbi.1003489.s005.tif]
