# Supplementary material for: Optimal Recall from Bounded Metaplastic Synapses: Predicting Functional Adaptations in Hippocampal Area CA3
Source: PLoS Comput Biol. 2014 Feb 27;10(2):e1003489. doi: 10.1371/journal.pcbi.1003489 (PMC3937414; doi:10.1371/journal.pcbi.1003489)
Supplement: Text S1 — Alternative recall dynamics. (PDF) [file pcbi.1003489.s006.pdf]

Supplementary Text S1. Alternative recall dynamics

Maximum a posteriori dynamics

Maximum a posteriori (MAP) dynamics dynamics are deterministic, implementing coordinate ascent to a potentially local maximum of the posterior $\mathrm{P}(\mathbf{x}|\mathbf{W}, \tilde{\mathbf{x}})$. For the posterior considered above (Eq. 18), coordinate ascent results in MAP dynamics that are very similar to those of the Gibbs sampler, with the distinction that the transfer function becomes a step function (Fig. S1B).

Mean-field dynamics

A third class of dynamics can be obtained using a mean-field formalism, in which the neurons have analog activations μ_i parametrizing a probability distribution over patterns $\mathbf{x}$ as a set of independent Bernoulli random variables:

$$\mathrm{Q}(\mathbf{x}; \boldsymbol{\mu}) = \prod_i \mu_i^{x_i} (1 - \mu_i)^{1-x_i} \tag{29}$$

In this framework, the objective for the recall dynamics is to optimize the parameters $\boldsymbol{\mu}$ to bring the represented distribution as close as possible to the true posterior, $\mathrm{P}(\mathbf{x}|\tilde{\mathbf{x}}, \mathbf{W}, \mathbf{C})$, in particular, by minimizing the Kullback-Leibler divergence between the two:

$$\mathrm{KL}\left[\mathrm{Q}(\mathbf{x}; \boldsymbol{\mu}) \| \mathrm{P}(\mathbf{x}|\tilde{\mathbf{x}}, \mathbf{W}, \mathbf{C})\right] = \sum_{\mathbf{x}} \mathrm{Q}(\mathbf{x}; \mathbf{W}, \tilde{\mathbf{x}}) \log \frac{\mathrm{Q}(\mathbf{x}; \mathbf{W}, \tilde{\mathbf{x}})}{\mathrm{P}(\mathbf{x}|\tilde{\mathbf{x}}, \mathbf{W}, \mathbf{C})} \tag{30}$$

Since the normalizing constant $\mathrm{P}(\tilde{\mathbf{x}}, \mathbf{W}|\mathbf{C})$ does not depend on $\mathbf{x}$, minimizing this distance is equivalent to optimizing the free energy [30]:

$$\mathrm{F}(\boldsymbol{\mu}, \tilde{\mathbf{x}}, \mathbf{W}, \mathbf{C}) = \sum_{\mathbf{x}} \mathrm{Q}(\mathbf{x}; \boldsymbol{\mu}) \log \mathrm{Q}(\mathbf{x}; \boldsymbol{\mu}) - \sum_{\mathbf{x}} \mathrm{Q}(\mathbf{x}; \boldsymbol{\mu}) \log \mathrm{P}(\mathbf{x}, \tilde{\mathbf{x}}, \mathbf{W}|\mathbf{C}) \tag{31}$$

Knowing that $\mathrm{P}(\mathbf{x}, \tilde{\mathbf{x}}, \mathbf{W}|\mathbf{C}) = \mathrm{P}_{\mathrm{store}}(\mathbf{x}) \cdot \mathrm{P}_{\mathrm{noise}}(\tilde{\mathbf{x}}|\mathbf{x}) \cdot \mathrm{P}(\mathbf{W}|\mathbf{x}, \mathbf{C})$ (Eq. 18) and making use of the approximation that the distribution $\mathrm{P}(\mathbf{W}|\mathbf{x}, \mathbf{C})$ factorizes over elements of $\mathbf{W}$, and the fact that $\mathrm{Q}(\mathbf{x}; \boldsymbol{\mu})$ is factorized over pairs of elements from $\mathbf{x}$ and $\boldsymbol{\mu}$, we rewrite the free energy as:

$$\begin{aligned}\mathrm{F}(\boldsymbol{\mu}, \tilde{\mathbf{x}}, \mathbf{W}, \mathbf{C}) = &\sum_i \mu_i \log \mu_i + (1 - \mu_i) \log(1 - \mu_i) - \sum_i \mu_i \log\left(\frac{f}{1-f}\right) + 2\log(1-f) - \\ &- \sum_i \tilde{x}_i (2\mu_i - 1) \log\left(\frac{1-r}{r}\right) + \mu_i \log\left(\frac{r}{1-r}\right) + \log(1-r) - \sum_{ij: C_{ij}=1} f(W_{ij}, \mu_i, \mu_j)\end{aligned} \tag{32}$$

where we can express $f(W_{ij}, x_i, x_j)$ as a polynomial: $f(W_{ij}, x_i, x_j) = b_1 W_{ij} x_i x_j + b_2 W_{ij} x_i + b_3 W_{ij} x_j + b_4 x_i x_j + b_5 x_i + b_6 x_j + b_7 W_{ij} + b_8$, with the coefficients $b_{1\ldots7}$ uniquely determined by the learning rule and the prior over patterns. Taking the derivative and reordering the terms appropriately, we obtain:

$$\begin{aligned}\frac{\partial \mathrm{F}(\boldsymbol{\mu}, \tilde{\mathbf{x}}, \mathbf{W}, \mathbf{C})}{\partial \mu_i} = &\log\left(\frac{\mu_i}{1-\mu_i}\right) - \log\left(\frac{f}{1-f}\right) - 2\log\left(\frac{1-r}{r}\right)\tilde{x}_i - \log\left(\frac{r}{1-r}\right) - \\ &- \sum_{j: C_{ij}=1} a_1^{\mathrm{in}} \cdot W_{ij}\, x_j + a_2^{\mathrm{in}} \cdot W_{ij} + a_3^{\mathrm{in}} \cdot x_j + a_4^{\mathrm{in}} - \\ &- \sum_{j: C_{ij}=1} a_1^{\mathrm{out}} \cdot W_{ji}\, x_j + a_2^{\mathrm{out}} \cdot W_{ji} + a_3^{\mathrm{out}} \cdot x_j + a_4^{\mathrm{out}}\end{aligned} \tag{33}$$

where, importantly, the parameters $a_{1\ldots4}^{\mathrm{in/out}}$ are exactly the same as for the Gibbs dynamics derived above.

Finally, using coordinate descent to minimize $\mathrm{F}(\boldsymbol{\mu}, \tilde{\mathbf{x}}, \mathbf{W}, \mathbf{C})$ wrt. $\boldsymbol{\mu}$, i.e. setting $\frac{\partial\,\mathrm{F}(\boldsymbol{\mu}, \tilde{\mathbf{x}}, \mathbf{W}, \mathbf{C})}{\partial \mu_i} = 0$ and solving for μ_i to obtain asynchronous update dynamics for μ_i [30], results in dynamics that are closely related to those defining Gibbs sampling, derived above. Both have exactly the same expression for the total somatic current to a neuron, but with the distinction that the neural transfer function $\sigma(I_i) = \frac{1}{1+e^{-I_i}}$ directly defines the neuron's (analog) activation rather than its probability of firing (Fig. S1B). Furthermore, neural activities μ_i can be used to predict uncertainty at retrieval, in a very similar way as response variability for the sampling-based representation.

Retrieval performance for different representations of the posterior distribution

We have shown that different types of recall dynamics translate into the same expression for the total somatic current to a neuron, but with different transfer functions operating on that somatic current (Fig. S1B). This means that our predictions for the circuit motifs involved in implementing optimal recall are independent from the precise type of dynamics assumed at recall. However, these can still influence the final recall performance. For instance, we expect the deterministic MAP dynamics to get stuck in local optima, which would be detrimental for recall. Comparing the performance for exact recall using the three types of recall dynamics (Fig. S1C) indeed reveals slightly worse average performance for MAP, relative to that obtained with sampling, while mean-field dynamics do just as well.

It is also important to note that the different dynamics each optimize a different cost function. While the posterior mean computed in a sampling-based representation (or approximated in the mean-field solution) is guaranteed to be optimal when using the Euclidian distance for measuring errors (as we do here), the deterministic MAP dynamics are optimal when an L0 norm is used as the error function, i.e. the same cost is incurred whenever the pattern is not recovered exactly, regardless of how similar the retrieved pattern is to the original. While it is not perfectly clear which metric is the most relevant biologically, a graded error seems more reasonable, which justifies our choice.
