# Supplementary material for: Optimal Recall from Bounded Metaplastic Synapses: Predicting Functional Adaptations in Hippocampal Area CA3
Source: PLoS Comput Biol. 2014 Feb 27;10(2):e1003489. doi: 10.1371/journal.pcbi.1003489 (PMC3937414; doi:10.1371/journal.pcbi.1003489)
Supplement: Text S2 — Comparison to standard attractor dynamics. (PDF) [file pcbi.1003489.s007.pdf]

Supplementary Text S2. Comparison to standard attractor dynamics

Inspired by previous heuristic extensions of standard attractor dynamics to metaplastic synapses [38], we also constructed such dynamics and compared their performance to that obtained by our model. For this, we stored a set of $T = 10$ patterns, denoted by $\mathbf{x}^{(t)}$, with $t \in \{1 \dots T\}$, drawn from our usual prior with $f = 0.5$, in a fully connected network of $N = 500$ neurons, by first initializing weights at random according to their stationary distribution, and then by using the cascade rule (either the presynaptically- or the postsynaptically-gated variant) to modify synapses as a function of the patterns. For recall, we used a heuristic form for the current to a neuron [38]:

$$I_i = \sum_j W_{ij}\, x_j - \sum_j x_j + h_i, \tag{34}$$

and set the threshold h_i as a function of all the patterns to be retrieved using an offline procedure. Specifically, h_i was set to be at the half-distance between the mean current induced by the patterns in which the neuron is active or inactive, respectively. More formally, if $m_0 = \sum_{t:x_i^{(t)}=0} \left(\sum_j W_{ij} x_j^{(t)} - \sum_j x_j^{(t)}\right)$ and $m_1 = \sum_{t,:x_i^{(t)}=1} \left(\sum_j W_{ij} x_j^{(t)} - \sum_j x_j^{t}\right)$ are the mean currents expected to be received by neuron i for patterns in which its state should be 0 or 1, respectively, then $h_i = (m_0 + m_1)/2$. (This condition guarantees maximum separability once the dynamics reached the appropriate fixed point, but see also for a discussion below). Note that the recall cue, $\tilde{\mathbf{x}}$ drawn from the usual input distribution with $r = 0.2$, does not explicitly appear in the total current, as – in line with standard approaches – it was only used to initialize the system. Finally, we used a step-function as the transfer function determining the output of the neuron, just as in our MAP dynamics (Text S1).

Unlike our model, the heuristic dynamics described above implicitly assume a uniform distribution over pattern ages, and there is no obvious principled way to incorporate a more realistic decaying prior over pattern ages in the dynamics. Thus, we simply plot the recall performance as a function of pattern age, without averaging the errors across it (Fig. S3; solid lines). For comparison, we also plot the performance of our optimal dynamics (Fig. S3; dashed lines). The quality of retrieval for the heuristic dynamics is remarkably poor, and in particular much poorer than for our dynamics, with errors above control for all but the most recent patterns. Worse still, even the average error would be above control for almost any prior for the pattern ages. There are several factors that contribute to this:

- Unlike our model, the heuristic dynamics uses the recall cue only as the initial condition, and ignores this source of information after that. This is detrimental for old memories, for which there is no information in the synaptic weights, so the only useful information is the recall cue (and the prior over patterns, when it is unbalanced). As a result, error rates for the heuristic dynamics saturate at the maximum possible value for a system that only has binary outputs ($\sqrt{1/2} \approx 71\%$). This supports the importance of using external fields for memory retrieval [8].
- The heuristic model has also no regulation of excitability depending on the weights of outgoing synapses, similar to $\mathrm{IP}_{\mathrm{out}}$ in our model, which was crucial for ensuring competent recall for postsynaptically-gated learning rules (Fig. 4A, top). In fact, ignoring the information stored in outgoing synapses in this way roughly corresponds to the simplest approximation of the evidence from outgoing synapses in our model, which – as we described in the main text – was a markedly efficient approximation. Consequently, retrieval is particularly poor when storage is postsynaptically-gated (red line).
- Although the setting of the threshold h_i, the analogue of $\mathrm{IP}_{\mathrm{in}}$ in our model, is guaranteed to work at a fixed point, it does not guarantee that starting from the recall cue, which is different from the pattern that needs to be recalled by definition, the dynamics would actually converge to the correct answer (formally, there is no guarantee on the size of the corresponding basin of attraction). The kind of off-line threshold regulation used here has another key shortcoming – though mostly of biological rather than computational relevance: it seems difficult to implement it in a neural circuit, whereas we have shown that standard models of IP can well approximate the optimal threshold setting in our model.

- The evolution of the network is deterministic, rather than stochastic. Although, as we showed above (Text S1), stochastic dynamics in our system was indeed able to achieve better performance than (binary) deterministic dynamics (implementing MAP inference), this advantage was only marginal. Therefore, while using binary deterministic dynamics can have a detrimental effect, we expect that this is not the main cause of the significantly poorer performance of the heuristic dynamics.

- One potential caveat in the comparison of the retrieval performance of the optimal versus heuristic dynamics has to do with a subtle difference in the way multiple patterns were stored in them. For the 'heuristic' network presented here, all patterns were stored in the synapses, while in for our optimal network we only stored one pattern and simulated the effects of storing the others independently in each synapse. In this way, we allowed our model to sidestep the problem caused by synaptic correlations [43], while the heuristic model had to cope with this additional difficulty. Therefore, as a further control, we also constructed an analog of our 'pseudo-storage' procedure for the heuristic network to ensure that it was also unaffected by synaptic correlations. However, for the specific parameters used here, this still did not improve its performance. Therefore, it is the heuristic form of the standard dynamics, and not other factors, that is mainly responsible for its poor performance.
