# Supplementary material for: Optimal Recall from Bounded Metaplastic Synapses: Predicting Functional Adaptations in Hippocampal Area CA3
Source: PLoS Comput Biol. 2014 Feb 27;10(2):e1003489. doi: 10.1371/journal.pcbi.1003489 (PMC3937414; doi:10.1371/journal.pcbi.1003489)
Supplement: Text S3 — Error rates for old patterns. (PDF) [file pcbi.1003489.s008.pdf]

Supplementary Text S3. Error rates for old patterns

The error rate for very old patterns becomes worse than that of the control network (Fig. 2C, blue). This is not unexpected, because we constructed network dynamics to optimise *average* recall performance, where the average is taken jointly over patterns and their ages. For deriving such optimal dynamics, the (age-)averaged contribution of a pattern to the synaptic weights needed to be taken into account (Methods). This average is smaller than the actual contribution for recent memories, but larger for old memories. Both mismatches lead to performance loss (compared to the hypothetical ideal case in which pattern age would be known, Fig. 2C, black), but in unequal measure. For recent memories, it only means that the system is not drawn strongly enough towards the correct pattern. However, it is particularly damaging for remote memories, as instead of the system being drawn towards the appropriate pattern, it is drawn towards a random other pattern.
